# Metabolism of alcohol ethoxylates (AEs) in rat, hamster, and human hepatocytes and liver S9: a pilot study for metabolic stability, metabolic pathway, and metabolites identification in vitro and in silico

**DOI:** 10.1007/s00204-024-03761-y

**Published:** 2024-06-06

**Authors:** Quan Shi, Stefan Moors, James Dawick, Lauren Kavanagh, Theresa Neely, Yuan Tian, Birte Dreeßen, Juan-Carlos Carrillo, Holger Hein, Peter J. Boogaard

**Affiliations:** 1 Shell Global Solutions International B.V., Carel van Bylandtlaan 16, 2596 HR The Hague, The Netherlands; 2 BASF Personal Care and Nutrition GmbH, Henkelstrasse 67, 40589 Düsseldorf, Germany; 3 Innospec Limited, Innospec Manufacturing Park, Oil G Sites Road Ellesmere Port, Cheshire, CH65 4EY UK; 4 Dr. Knoell Consult Ltd., 22 Cathedral Road, Cardiff, CF11 9IJ UK; 5 Institute of Ophthalmology, University College London, 11-43 Bath St, London, EC1V 9EL UK; 6 Sasol Germany GmbH, Paul-Baumann-Str. 1, 45772 Marl, Germany; 7 Knoell Germany GmbH, Marie-Curie-Straße 8, 51377 Leverkusen, Germany; 8 Division of Toxicology, Wageningen University and Research, Stippeneng 4, 6708 WE Wageningen, The Netherlands

**Keywords:** Alcohol ethoxylates (AEs), Hepatocytes, Liver S9, OECD QSAR toolbox, Biotransformation pathways, Metabolites profiles

## Abstract

Alcohol ethoxylates (AEs) are a well-known class of non-ionic surfactants widely used by the personal care market. The aim of this study was to evaluate and characterize the in vitro metabolism of AEs and identify metabolites. Five selected individual homologue AEs (C_8_EO_4_, C_10_EO_5_, C_12_EO_4_, C_16_EO_8_, and C_18_EO_3_) were incubated using human, rat, and hamster liver S9 fraction and cryopreserved hepatocytes. LC–MS was used to identify metabolites following the incubation of AEs by liver S9 and hepatocytes of all three species. All AEs were metabolized in these systems with a half-life ranging from 2 to 139 min. In general, incubation of AE with human liver S9 showed a shorter half-life compared to rat liver S9. While rat hepatocytes metabolized AEs faster than human hepatocytes. Both hydrophobic alkyl chain and hydrophilic EO head group groups of AEs were found to be target sites of metabolism. Metabolites were identified that show primary hydroxylation and dehydrogenation, followed by O-dealkylation (shortening of EO head groups) and glucuronidation. Additionally, the detection of whole EO groups indicates the cleavage of the ether bond between the alkyl chain and the EO groups as a minor metabolic pathway in the current testing system. Furthermore, no difference in metabolic patterns of each individual homologue AE investigated was observed, regardless of alkyl chain length or the number of EO groups. Moreover, there is an excellent agreement between the in vitro experimental data and the metabolite profile simulations using in silico approaches (OECD QSAR Toolbox). Altogether, these data indicate fast metabolism of all AEs with a qualitatively similar metabolic pathway with some quantitative differences observed in the metabolite profiles. These metabolic studies using different species can provide important reference values for further safety evaluation.

## Introduction

Alcohol ethoxylates (AEs) are a major class of non-ionic surfactants that are commonly manufactured and utilized by many industrial practices and commercial markets (Sanderson et al. 2013). These compounds are synthesized via the reaction of fatty alcohols and ethylene oxide, resulting in a molecule that contains a hydrophobic alkyl chain attached via an ether linkage to hydrophilic ethylene oxide (EO) groups. Due to the amphiphilic structure of AEs, where a molecule can inhabit the interface of two immiscible phases (i.e. oil and water) and effectively bring them closer together, AEs are widely used in laundry and dishwasher detergents and to a lesser extent in household cleaners, institutional and industrial cleaners, cosmetics, agriculture, and in textile, paper, oil and other process industries (HERA 2009). AEs have the general structure: R(OCH_2_CH_2_)_*n*_OH; where R is the alkyl chain which can vary in length and in the degree of linearity. AEs are also typically defined as “C_*x*_EO_*n*_” where the subscript *x* following the ‘*C*’ indicates the range of carbon chain units, and typically between 8 and 18 carbons long (for detergent range surfactants) (HERA 2009). The subscript n following the ‘EO’ indicates the degree of ethoxylation, which can also vary in length from 1 to 40 EO groups (fatty alcohol are the special case to the formula where *n* = 0, C_*x*_EO_0_) (Sanderson et al. 2013). For example, an AE with the structure C_10_EO_5_ contains an alkyl chain length of 10 carbon atoms and a side chain composed of 5 EO groups. In addition, during the manufacturing, the ethoxylation process leads to a distribution of EO units attached to each alkyl chain resulting in complex technical mixtures. For instance, C_9–11_EO_2.5_, which contains a range of alkyl chain lengths of 9–11 and averages 2.5 EO units per alkyl chain (full EO range typically being EO_0_-EO_14_ but distribution peaks at 2–3 mol EO).

The evaluation of absorption, distribution, metabolism, and excretion (ADME) properties of chemicals plays a useful role in providing insights into the relevant toxicological properties of a compound which are important for toxicity interpretation in human risk assessment (Barton et al. 2006; Schroeder et al. 2011; WHO 2009). Typically, data on chemical metabolism and toxicokinetics generated during early hazard assessment include metabolic stability (rate of metabolism), potential metabolic pathways, and metabolite identification (Prasad et al. 2011). Overall, the information obtained not only serves as an adequate basis for hazard characterization related to the active chemical entity in the circulation or tissue, but can also provide essential information to underpin grouping AEs and applying the read-across defined by European Chemicals Agency (ECHA) “*Read-across Assessment Framework*” (RAAF) (ECHA 2017).

The ADME of AEs has been extensively studied in vivo in both rats and human volunteers (Drotman 1980; HERA 2009; Talmage 1994; Unilever 1978). In a study, female Colworth Wistar rats were orally administrated with three ^14^C-labelled AEs (i.e. C_12_EO_3_, C_12_EO_6_, and C_12_EO_10_), and placed in a metabolism chamber for 4 days while feces, urine, air, and various tissues and organs were monitored for ^14^C activity. In this study, the total recovery in urine, feces, air, and carcass of the administered compound was close to 100%, and ^14^C was excreted mainly in the urine (about 10% ^14^C in air) after oral administration (Unilever 1978). In another study, elimination and resorption of ^14^C-labelled C_14–18_EO_10_ were monitored over 72 h after a single oral gavage application to Wistar rats. Approximately 90% of the compound was excreted within the first 24 h and about 98–99% of the compound was eliminated within 72 h. Again, the majority of the administrated compound was excreted in the urine and in the feces, and about 2% was excreted as ^14^CO_2_ in air. In a human volunteer study, six adult males (bodyweight 60–90 kg) per treatment group were given a capsule containing 50 mg of the radio-labelled surfactant (i.e. ^14^C-labelled C_12_EO_6_ and C_13_EO_6_ labelled in the carbon chain or ethoxy chain), and their blood, urine, feces, and expired CO_2_ were collected (Drotman 1980). Most of the radioactivity (i.e. about 83–89%) for both compounds was recovered after 144 h in urine, feces, and air while the amounts in the blood were very low and never exceeded 1%.

The Human and Environmental Risk Assessment on ingredients of European household cleaning products report summarized all relevant ADME studies from AEs and concluded that the metabolism of AE is shown to be rapid and complete (HERA 2009). Meanwhile, the report also hypothesized that the major biotransformation pathway of AEs appears to be the hydrolysis of the ether linkage and subsequent oxidation of the resulting alcohol to fatty acids which are degraded to C_2_-fragments and shorter alkyl chains and ultimately to carbon dioxide and water. The other lower molecular weight polyethylene glycol (PEG) by-products from primary metabolism are further degraded by breakdown of the ether linkage or are excreted via urine (HERA 2009). Moreover, studies with radio-labelled compounds showed that both the alkyl chain and the EO groups are sites of attack. Thus, the PEG materials will also be degraded to varying C chain lengths.

Despite extensive studies on the absorption and excretion of AEs, very little is known about the comparability of the metabolism pathway, kinetic constancy, and potential metabolites in different species. Hence, to investigate this further and gather information about the comparability of metabolite patterns/parameters in different species and with different AEs underlining the hypothesis within the HERA 2009 assessment, in vitro metabolism (phase I and phase II) studies on five individual homologue AEs (i.e. C_8_EO_4_, C_10_EO_5_, C_12_EO_4_, C_16_EO_8_ and C_18_EO_3_) using rat, hamster and human liver S9 and cryopreserved hepatocytes was performed. Within this pilot study, information on the metabolic stability, metabolites, biotransformation pathways, and concluding toxicokinetic parameters of AEs was collected. In addition, in silico quantitative structure–activity relationship (QSAR) modelling using the OECD QSAR Toolbox (version 4.5) was used to simulate the metabolic fate of AEs for comparison with the experimental in vitro studies.

## Materials and methods

### Chemicals and suppliers

HPLC-grade methanol and acetonitrile: Merck (Darmstadt, Germany). HPLC-grade formic acid, acetic acid, ammonium acetate, and ammonium formate: BDH Laboratory Supplies (Poole, UK). Other chemicals: Sigma Aldrich (Helsinki, Finland), the highest purity available. Water was in-house freshly prepared with a Direct-Q3 (Millipore Oy, Espoo, Finland) purification system and UP grade (ultrapure, 18.2 MΩ). The study compounds were purchased from Sigma-Aldrich and described in Table 1.

**Table 1:** chemical name and properties of each test item

Abbreviation name	CAS no.	Sigma number	Lot	MW	Full name	Purity (%)
C_8_EO_4_	19327-39-0	T3394	BCCF1360	306.44	Tetraethylene glycol monooctyl ether	≥ 98
C_10_EO_5_	23244-49-7	76436	BCCB9565	378.54	Pentaethylene glycol monodecyl ether	≥ 97
C_12_EO_4_	5274-68-0	1372424	FOJ132	362.54	Tetraethylene glycol monododecyl ether	≥ 99
C_16_EO_8_	5698-39-5	74717	BCCF0615	594.86	Octaethylene glycol monohexadecyl ether	≥ 98
C_18_EO_3_	4439-32-1	AS-3199	Bx 96596	402.65	Triethylene glycol monooctadecyl ether	≥ 99
		KEY465201438				

### Incubation materials and procedures for liver S9 fraction

The metabolic stability assay was performed in a 48-well plate format (duplicate with cofactors and single without cofactors). The pooled liver S9 from three species (i.e. human, hamster, and rat) were purchased from Bioreclamation IVT (see “Appendix 1” Table 9 for a detailed description). Results (half-lives) for disappearance control midazolam are shown in “Appendix 1” Table 11, showing that enzyme activities were at a normal level.

Each incubation contained test compound (final concentration of 1 or 10 µM), liver S9 (1.5 mg/ml protein content), Cofactors (1 mM NADPH + 1 mM UDPGA + 0.2 mM PAPS), MgCl_2_ (2 mM), and potassium phosphate buffer (100 mM at pH 7.4). The final incubation volume was 300 µl containing 0.5% DMSO as solvent for all the AEs or 0.25% DMSO + 0.25% IPA for C_18_EO_3_. The incubation was carried out at 37 °C for 60 min with and without cofactors. At each time point (0, 5, 10, 20, 40, and 60 min), the reactions were quenched with twofold volume of 75% acetonitrile. The samples were collected and stored at − 20 °C for further analysis. Midazolam (1 µM) was used as a disappearance control for determining the disappearance rate.

### Incubation materials and procedures for hepatocytes

The metabolic stability assay was performed in a 48-well plate format (duplicate with cells and single without cells). The pooled cryopreserved hepatocytes from three species/strain (i.e. human, hamster, and rat) were purchased from Bioreclamation IVT (see “Appendix 1” Table 10 for a detailed description). Results (half-lives) for disappearance of control verapamil are shown in “Appendix 1” Table 12, showing that enzyme activities were at a normal level.

The hepatocytes were thawed and re-suspended in Celsis InVitro KHB medium (pH 7.4). Cell count and cell viability (i.e. Human viability 86%, Hamster viability 69%, and Rat viability 62%) were determined by Trypan Blue exclusion method. The final incubation volume was 320 µl containing 1 million viable cells/ml and 0.5% DMSO (0.25% DMSO + 0.25% IPA for C_18_EO_3_) with test compounds achieving a final concentration of 1 or 10 µM. The incubation (with and without cells) was carried out at 37 °C for 120 min with shaking (600 rpm). At each time point (0, 5, 15, 30, 60 and 120 min), the reactions were quenched with twofold volume of 75% acetonitrile. The samples were collected and stored at − 20 °C for further analysis. Verapamil (1 µM) was used as a disappearance control for determining the disappearance rate.

### Analytical methods for metabolites profiling in hepatocytes and liver S9

The samples were thawed at room temperature (RT), shaken, and centrifuged for 20 min at 2272 × g (Thermo SL16, room temperature), and pipetted to Waters 96-well UPLC-plate for analysis.

The in vitro metabolism of the five individual homologue AEs (i.e. C_8_EO_4_, C_10_EO_5_, C_12_EO_4_, C_16_EO_8_, and C_18_EO_3_) was analyzed by liquid chromatography–mass spectrometry (LC–MS). The UPLC-Q-Exactive Orbitrap MS system consisted of a Thermo Vanquish Horizon UHPLC with an autosampler, vacuum degasser, photodiode array (PDA) detector (210–500 nm), and column oven coupled to a Q-Exactive Orbitrap Focus mass spectrometer (Thermo Fisher Scientific, Waltham, MA, USA). The analytical column used was a Waters Acquity BEH C8 2.1 × 50 mm with 1.7 particle size (Waters Corp, Milford, MA, USA). The temperature of the column oven was 40 °C, and the injection volume was 4 μl. The aqueous eluent (A) was 0.1% formic acid (B) was acetonitrile. A gradient elution with 98–98–(100-*X*)–2–2–98% (B) in 2–2-*X*–98–98–2 min (*X* = 60 for C_8_EO_4_, 80 for C_10_EO_5_ and C_12_EO_4_, 98 for C_16_EO_8_ and C_18_EO_3_) was applied, followed by 1 min equilibration time. The eluent flow rate was 0.5 ml/min and the flow was directed to the MS through a PDA detector. The data acquisition was performed using positive Electrospray Ionization (ESI+) polarity with a capillary voltage of 3000 V. Capillary temperature was 320 °C and auxiliary gas temperature was 500 °C. The mass spectrometer was operated in the data-dependent MS2 mode, which acquires full-scan MS and MS/MS fragment ion data in the same run. Scan was performed with a resolution of 35,000 (full width at half maximum at m/z 200), while an Automated Gain Control target of a million ions, maximum injection time of 100 ms, and a scan range of 100–1000 m/z were used. Resolution of 17,500 (full width at half maximum at m/z 200) and collision energies of 20, 40, and 60 eV were used in the ddMS^2^ mode. Nitrogen was used as a sheath gas with 50 units, auxiliary gas with 10 units and as a sweep gas with 5 units. Ion chromatograms were extracted from the total ion chromatograms using calculated monoisotopic accurate masses with 5 mDa window. Calibration curves were generated using an external standard. The data were processed with Thermo Xcalibur 4.1.31.9 software.

### Half-life and clearance calculations

The first-order rate constants *k* (min^−1^) of the metabolism were obtained from the slope of time versus ln (% remaining) plots using Excel software. All time points (log-scale) were used in fitting the rate constant *k* based on visual inspection of the curves.

The in vitro half-life (*t*1/2) of the test compound(s) is defined as:

$$t_{1/2} = \ln 2/k$$

Intrinsic in vitro clearance was calculated as follows:

CL_int,inc_ = k*V/(*M*), where *V* is the volume of the incubation and *M* is the number of cells or amount of S9 protein in the incubation.

### In silico OECD QSAR Toolbox prediction

The potential metabolites of the five individual homologue AE substances were predicted using the OECD QSAR Toolbox (version 4.5) (https://qsartoolbox.org). The metabolism and transformation simulators used to identify potential metabolites are:

- Hydrolysis (acidic)
- In vivo Rat metabolism
- Rat liver S9 metabolism
- Skin metabolism

The simulation of metabolism and transformation was performed for each individual AE homologue using Simplified Molecular Input Line Entry System (SMILES) codes as input to the model. Results of the in silico metabolic simulations with OECD QSAR toolbox are summarized in “Appendix 1” Table 18.

## Results

### Metabolic stability in liver S9 fraction

**Fig. 1:**
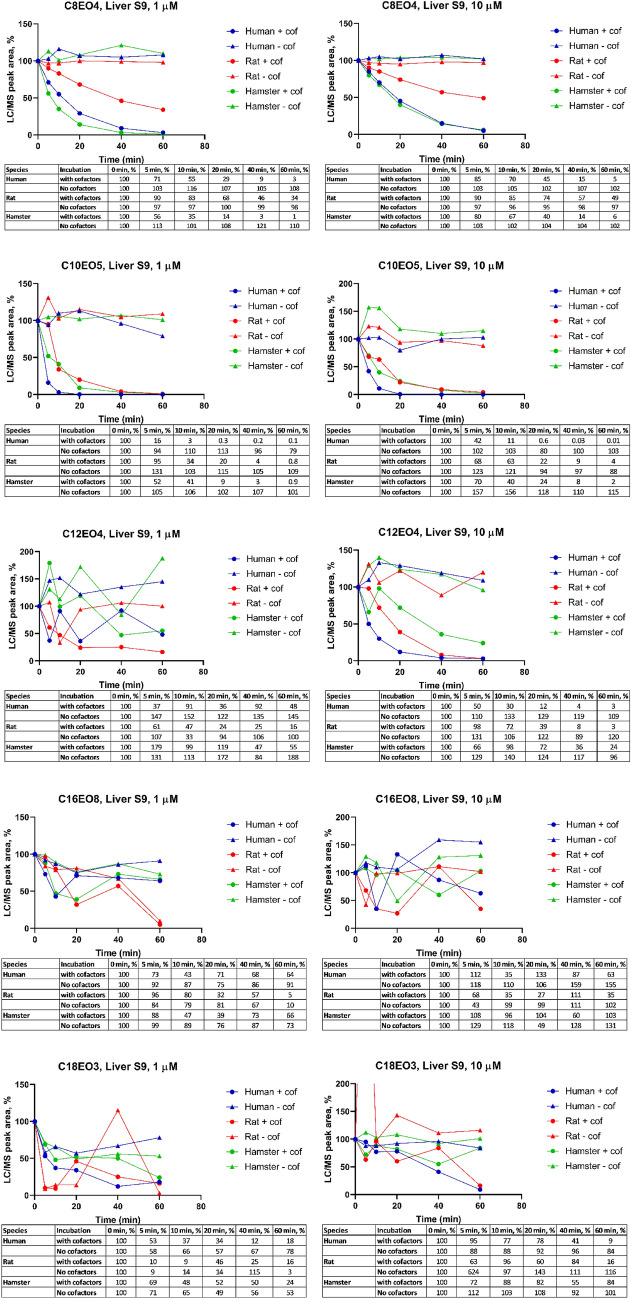
Relative LC/MS peak areas for C_8_EO_4_, C_10_EO_5_, C_12_EO_4_, C_16_EO_8_, and C_18_EO_3_ in investigated time points with initial concentration of 1 and 10 µM and liver S9 fraction concentration 1.5 mg/ml, with cofactor (*n* = 2) and without cofactors (*n* = 1)

For each AE, the relative LC/MS peak areas with and without cofactors in liver S9 fraction of the investigated species (i.e. human, rat, and hamster) over the 60 min time period are shown in Fig. 1. In general, a cofactor-dependent disappearance was observed for all investigated compounds, being most apparent with C_8_EO_4_, C_10_EO_5_, and C_12_EO_4_ whilst no disappearance was observed without cofactors. At a concentration of 1 and 10 µM C_8_EO_4_, only 1–6% of the initial concentration was remaining after 60 min incubation with human and hamster liver S9, while the corresponding value in rat was 34–49%. At a concentration of 1 and 10 µM C_10_EO_5_, only 0.01–4% of the initial concentration was remaining after 60 min incubation with human, rat, and hamster liver S9. The disappearance of C_12_EO_4_ showed high fluctuation in LC/MS peak areas at 1 μM, while the quality of data from 10 μM incubations was substantially better. At a concentration of 10 μM, the remaining abundances after 60 min incubation were 3% for human, 3% rat, and 24% for hamster. A high fluctuation of LC/MS peak areas was observed in the results with C_16_EO_8_ and C_18_EO_3_ at both high and low concentrations which hampered further investigation on these two individual AE homologues.

### Metabolic stability in hepatocytes

**Fig. 2:**
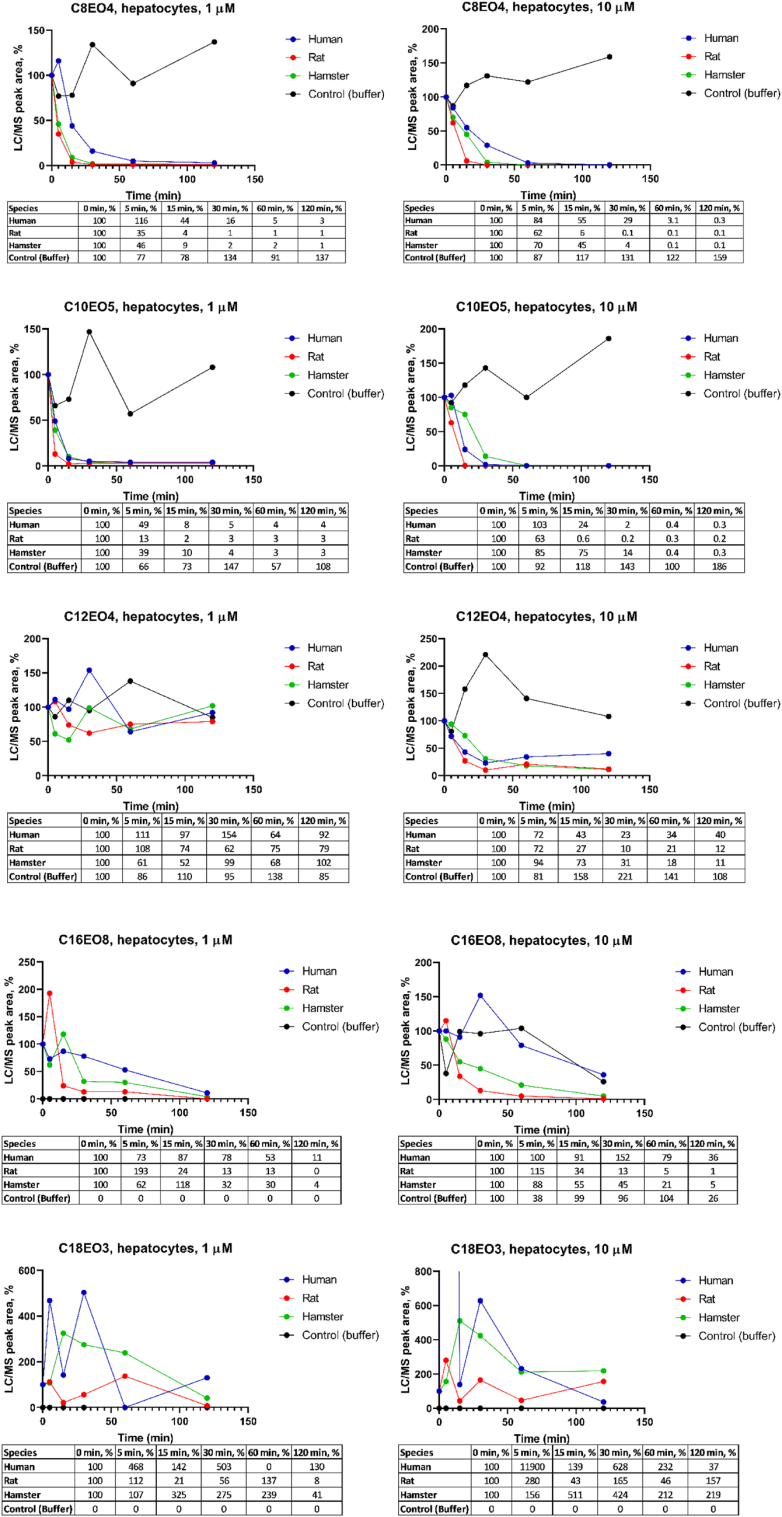
Relative LC/MS peak areas for C_8_EO_4_, C_10_EO_5_, C_12_EO_4_, C_16_EO_8_ and C_18_EO_3_ in investigated time points with initial concentration of 1 and 10 μM and hepatocytes content of 1 million/ml (*n* = 2)

The results from incubation with human, rat, and hamster hepatocytes and without hepatocytes are shown in Fig. 2. In general, no disappearance was observed in incubations without cells, but high variation in the peak areas was observed with all compounds, including no detection of test item (C_16_EO_8_ at 1 μM and C_18_EO_3_ for both concentrations) in buffer incubations. At a concentration of 1 and 10 µM C_8_EO_4_, only 0.1–3% of the initial concentration was remaining after 120 min incubation with human, rat, and hamster hepatocytes. At a concentration of 1 and 10 µM C_10_EO_5_, only 0.2–4% of the initial concentration was remaining after 120 min. Similar to results with the liver S9 incubations, the disappearance of C_12_EO_4_ at a concentration of 1 μM showed high fluctuation in LC/MS peak areas. However, the data from 10 μM incubations with this AE homologue was of good quality and the incubations at 1 µM were not subject to further investigation. At a concentration of 10 μM, the remaining C_12_EO_4_ after 120 min incubation was 40% for human, 12% for rat and 11% for hamster. At a concentration of 1 and 10 µM C_16_EO_8_, 11–36%, 1–16%, and 4–5% of the compound was remaining after 120 min in human, rat, and hamster hepatocytes, respectively. A high fluctuation of LC/MS peak areas was observed in the data of C_18_EO_3_, at both test concentrations which hampered further investigation efforts.

### Half-life and clearance calculations

Based on the disappearance data (Figs. 1, 2), half-lives and in vitro clearances were calculated and are presented in Table 2. Due to some poor-quality data shown by fluctuation of LC/MS peak areas as a function of incubation time, kinetic calculations were not able to be performed and are therefore shown as Not Available (NA) (see Table 2).

**Table 2:** Kinetic in vitro calculations based on the disappearance data in liver S9 and hepatocytes (*n* = 2)

Compounds	Test system	Concen.	Half-lives *t*1/2 (min)			In vitro CLint (μl/min/mg)		
			Human	Rat	Hamster	Human	Rat	Hamster
C_8_EO_4_	Liver S9	1 μM	11.8	37.8	7.7	39.2	12.2	59.7
		10 μM	13.8	58.1	14.5	33.6	7.95	31.8
	Hepatocyte	1 μM	12.9	3.2	4.3	53.7	220	160
		10 μM	12	3.6	6.5	57.7	191	107
C_10_EO_5_	Liver S9	1 μM	2.0	8.3	8.1	234	55.7	57.3
		10 μM	2.7	12	12	170	38	40
	Hepatocyte	1 μM	4.0	2.9	4.5	172	236	153
		10 μM	4.9	1.9	10.6	140	357	65.4
C_12_EO_4_	Liver S9	1 μM	NA	26	NA	NA	17.5	NA
		10 μM	9.0	11	26	51.2	40.7	17.9
	Hepatocyte	1 μM	NA	NA	789	NA	NA	0.88
		10 μM	15	8.9	17	47.4	78.0	39.7
C_16_EO_8_	Liver S9	1 μM	139	16	118	3.33	29.6	3.92
		10 μM	NA	NA	NA	NA	NA	NA
	Hepatocyte	1 μM	39	6.6	27	17.6	104	25.7
		10 μM	85	9.2	30	8.13	75	23.4
C_18_EO_3_	Liver S9	1 μM	29	32	29	15.9	14.6	16.2
		10 μM	NA	NA	NA	NA	NA	NA
	Hepatocyte	1 μM	NA	NA	NA	NA	NA	NA
		10 μM	NA	NA	NA	NA	NA	NA

*NA* not available

The majority of the AEs were metabolized within the incubation time in both liver S9 and hepatocytes. Half-lives for human liver S9 fraction and hepatocytes are comparable for C_8_EO_4_ and C_10_EO_5_ and ranged from 2 min in liver S9 and 4 min in hepatocytes for C_10_EO_5_ to ≥ 29 min for C_18_EO_3_ in liver S9. The majority of the half-lives were less than 30 min for most systems and test compounds. The outlier values might possibly be due to low concentrations of residual parent AE compounds and inherent analytical fluctuations.

In addition, the interpretation of data with AE with longer alkyl carbon chain lengths (> C12) was more problematic compared to the shorter C chain homologues. It seems that these higher molecular weight homologues are harder to detect or less metabolism could occur, which is observed by fluctuating data in C_12_EO_4_ (rat and hamster liver S9 at 1 μM, human and rat hepatocyte at 1 μM), C_16_EO_8_ (all species liver S9 at 10 μM) and C_18_EO_3_ (all species liver S9 at 10 μM and all hepatocyte). AEs (except C_16_EO_8_) were metabolized faster in human liver S9 (approx. 1.1–4.4 times) than rat liver S9 at both concentrations. All AEs were metabolized faster in rat (approx. 1.4–9.2 times) when compared with data from incubations with human hepatocytes.

### Metabolite identification for C_8_EO_4_

In liver S9 fraction, 20 metabolites (M1–M20) were detected across all species for C_8_EO_4_ (Table 3 and “Appendix 1” Table 13). In human S9 fraction, 17 metabolites were detected, with octane hydroxylation M2 (54%) being the most abundant metabolite, followed by octane di-hydroxylation and dehydrogenation M9 (16.1%) and ethoxy hydroxylation and dehydrogenation M8 (10.6%). In rat S9 fraction, 16 metabolites were detected, with M2 (26.6%) being clearly the most abundant metabolite, followed by M8 (5.8%) and octane hydroxylation M1 (4.6%). In hamster S9 fraction, 20 metabolites were detected with M1 (17.7%), M2 (34.3%), and hydroxylation in octane (M3, 9.7%) being the most abundant.

**Table 3:** Metabolite profiles for C_8_EO_4_

Metabolites code	Liver S9 with cofactors, %^a^			Liver S9 without cofactors, %^a^			Hepatocytes, %^b^			
	Human	Rat	Hamster	Human	Rat	Hamster	Human	Rat	Hamster	Buffer
C_8_EO_4_	7.1	54.4	9.5	98.9	99.5	99.3	0.1	0.1	0.1	99.8
M1	1.1	4.6	17.7	–	–	–	0.3	0.2	1.7	–
M2	54.0	26.6	34.3	–	–	–	1.3	0.8	7.0	-
M3	–	–	9.7	–	–	–	0.2	0.7	5.1	–
M4	–	0.5	2.9	–	–	–	0.1	0.0	1.1	–
M5	–	–	0.4	–	–	–	–	–	1.1	–
M6	0.1	–	0.3	–	–	–	0.3	0.4	1.0	–
M7	1.8	0.03	4.7	–	–	0.01	1.4	3.1	31.4	–
M8	10.6	5.8	2.2	0.6	0.2	0.2	3.7	–	1.0	0.1
M9	16.1	0.3	4.2	–	–	–	81.4	66.1	25.1	0.01
M10	2.1	0.2	0.0	–	–	–	–	–	–	–
M11	0.3	0.5	1.4	0.01	–	0.01	0.1^c^	0.1^c^	0.3^c^	–
M12	0.6	0.2	1.0	0.01	0.01	–	0.0	0.0	0.2	–
M13	0.2	–	0.1	–	–	–	0.1	0.1	0.7	–
M14	1.0	3.1	2.0	0.2	0.08	0.1	0.4	1.2	0.3	0.07
M15	1.5	1.1	3.5	0.3	0.2	0.3	0.6	0.8	2.3	–
M16	0.4	0.2	0.7	0.03	0.01	0.02	–	–	–	–
M17	3.1	2.5	5.6	–	–	–	0.8	0.5	0.2	–
M18	0.1	0.3	0.7	–	–	–	–	–	–	–
M19	0.01	0.07	0.8	–	–	–	–	–	–	–
M20	0.4	0.2	0.2	–	–	–	0.4	5.1	0.3	–
M21	–	–	–	–	–	–	0.0	0.2	4.7	–
M22	–	–	–	–	–	–	0.1	0.2	2.2	–
M23	–	–	–	–	–	–	3.3	6.2	2.0	–
M24	–	–	–	–	–	–	1.2	1.7	7.2	–
M25	–	–	–	–	–	–	1.4	9.8	1.9	–
M26	–	–	–	–	–	–	2.8	2.8	3.5	–

^a^For liver S9 data, results were from 60 min in liver S9 and presented as percentages of the total peak area at 60 min time point from 10 µM incubation

^b^For hepatocyte data, results were from 120 min in hepatocytes and presented as percentages of the total peak area at 120 min time point from 10 µM incubation

^c^Data from 15 min sample

In hepatocytes, 22 metabolites were detected across all species (Table 3 and “Appendix 1” Table 13). 21 metabolites were detected in human hepatocytes and the main metabolite was M9 (81.4%), followed by hydroxylation and dehydrogenation in the ethoxy moiety (i.e. the EO ‘tail’) (M8, 3.7%), 3 × hydroxylations and 2 × dehydrogenations (M23, 3.3%) and O-dealkylation (hydroxylation and loss of C4H8O2) with 4 × hydroxylations + dehydrogenation (M26, 2.8%). In rat, 20 metabolites were detected and M9 (66.1%) was the most abundant, followed by O-dealkylation with 3 × hydroxylations and 2 × dehydrogenations (M25, 9.8%). In hamster, 22 metabolites were detected and the most abundant were hydroxylation and dehydrogenation in octane (M7, 31.4%) and M9 (25.1%).

Differences were observed for M10, M16, M18, and M19, which were detected in S9 fraction, but not in hepatocytes whereas M21–M26 were not detected in S9 fraction but were detected in hepatocytes.

### Metabolite identification for C_10_EO_5_

For C_10_EO_5_, 26 metabolites were detected in liver S9 (Table 4 and “Appendix 1” Table 14). All metabolites were detected in human, with decane di-hydroxylation and dehydrogenation M6 (56.5%) being the most abundant metabolite, followed by decane hydroxylation M1 (25.3%). In rat, M1 (70%) was the most abundant metabolite, followed by M5 (5.8%) and M6 (6.9%) formed via ethoxy hydroxylation and dehydrogenation. The most abundant metabolites in hamster were M1 (32.3%), M6 (16%), and M25 (12.7%) formed via O-dedecylation.

**Table 4:** Metabolite profiles for C_10_EO_5_

Metabolite code	Liver S9 with cofactors, %^a^			Liver S9 without cofactors, %^a^			Hepatocytes, %^b^			
	Human	Rat	Hamster	Human	Rat	Hamster	Human	Rat	Hamster	Buffer
C_10_EO_5_	0.03	7.2	3.6	97.7	98.2	97.9	0.3	0.8	0.7	98.7
M1	25.3	70.0	32.3	0.2	0.04	0.03	0.2	0.04	1.6	0.0
M2	0.6	1.1	0.5	–	–	–	0.1	0.5	0.6	–
M3	0.8	0.1	0.3	–	–	–	0.3	0.3	0.4	–
M4	5.1	0.4	6.4	–	–	–	0.3	0.02	4.1	0.01
M4b	0.02	0.2	1.2	–	–	–	–	–	–	–
M5	0.01	5.8	3.6	1.2	1.0	1.2	0.03	0.04	0.1	0.3
M6	56.5	6.9	16.0	0.1	0.03	0.01	53.3	60.9	31.2	–
M6b	0.3	1.2	0.2	–	–	–	–	–	–	–
M8	1.4	–	–	–	–	–	1.7	0.9	1.3	–
M9	0.01	0.4	0.3	0.04	0.07	0.03	0.01	0.02	0.02	0.02
M11	0.3	0.9	3.3	–	–	–	0.01	0.01	0.2	–
M13	0.7	0.2	1.7	–	–	–	0.6	1.1	1.1	–
M16	0.7	0.6	5.5	–	–	–	0.4	0.1	0.3	–
M17	2.7	0.6	0.7	–	–	–	0.3	0.6	0.8	–
M18	0.01	0.02	0.3	–	–	–	–	–	–	–
M19	0.08	0.3	0.6	–	–	–	–	–	–	–
M20	0.04	0.7	1.8	–	–	–	–	–	–	–
M21	0.2	0.02	0.4	–	–	–	–	–	–	–
M22	0.03	0.2	0.4	–	–	–	–	–	–	–
M23	0.1	0.02	0.4	–	–	–	–	–	–	–
M24	0.03	–	0.5	–	–	–	0.02	1.7	0.4	–
M25	3.2	2.0	12.7	0.4	0.3	0.5	2.1	2.7	10.1	0.6
M26	0.03	0.04	0.05	–	0.01	0.02	0.2	0.4	0.9	0.01
M27	0.9	0.3	2.8	0.1	0.04	0.06	0.6	0.9	2.3	0.03
M28	0.5	0.5	3.8	0.2	0.1	0.2	0.3	0.6	2.0	0.3
M29	0.3	0.3	1.0	0.1	0.03	0.06	0.02	0.1	0.2	0.003
M30	–	–	–	–	–	–	0.04	1.5	2.2	–
M31	–	–	–	–	–	–	0.8	0.02	0.1	–
M32	–	–	–	–	–	–	0.2	0.1	3.8	–
M33	–	–	–	–	–	–	10.6	5.0	8.1	–
M34	–	–	–	–	–	–	0.5	0.7	0.7	–
M35	–	–	–	–	–	–	3.7	0.8	0.1	–
M36	–	–	–	–	–	–	18.4	10.8	20.6	0.01
M37	–	–	–	–	–	–	1.4	0.7	0.2	–
M38	–	–	–	–	–	–	0.7	0.5	2.9	–
M39	–	–	–	–	–	–	2.8	8.1	3.1	0.01

^a^For liver S9 data, results were from 60 min in liver S9 and presented as percentages of the total peak area at 60 min time point from 10 µM incubation

^b^For hepatocyte data, results were from 120 min in hepatocytes and presented as percentages of the total peak area at 120 min time point from 10 µM incubation

In hepatocytes in all species, 28 metabolites were detected for C_10_EO_5_ (Table 4 and “Appendix 1” Table 14). All metabolites in hepatocytes were detected in human and clearly the most abundant was M6 (53.3%), followed by O-dealkylation (loss of C_6_H_12_O_3_ with 5 × hydroxylations and dehydrogenation (M36, 18.4%) and O-dealkylation with 3 × hydroxylations and dehydrogenation in decane (M33, 10.6%). All metabolites were detected in rat, and M6 (60.9%) was clearly the main metabolite, followed by M36 (10.8%), O-dealkylation (loss of C_8_H_18_O_4_ with 6 × hydroxylations and dehydrogenation (M39, 8.1%) and M33 (5%). All metabolites were detected in hamster as well, and the most abundant metabolite was M6 (31.2%), followed by M36 (20.6%), Loss of C_10_H_20_ (M25, 10.1%), and M33 (8.1%).

M4b, M6b, and M18–M23 were detected in S9 fraction but were not detected in hepatocytes. M30–M38 were detected in hepatocytes but not in liver S9 fraction.

### Metabolite identification for C_12_EO_4_

In liver S9 fraction, a total of 20 metabolites were detected across all species for C_12_EO_4_ (Table 5 and “Appendix 1” Table 15). The most abundant metabolites in all species were ethoxy hydroxylation and dehydrogenation M4 (18.5% in human, 21.4% in rat, and 12.9% in hamster liver S9), dodecane di-hydroxylation and dehydrogenation M5 (30.1% in human, 16.4% in rat, and 11.9% in hamster liver S9), and O-dealkylation with dodecane di-hydroxylation and dehydrogenation M10 (23.7% in human, 18.6% in rat, and 16% in hamster liver S9). Additionally, M1 (14.5%) formed via dodecane hydroxylation had about similar abundance than M4, M5 and M10 in rat.

**Table 5:** Metabolite profiles for C_12_EO_4_

Metabolite code	Liver S9 with cofactors, %^a^			Liver S9 without cofactors, %^a^			Hepatocytes, %^b^			
	Human	Rat	Hamster	Human	Rat	Hamster	Human	Rat	Hamster	Buffer
C_12_EO_4_	2.1	2.0	18.1	98.8	99.7	98.4	12.5	12.7	13.2	79.1
M1	0.4	14.5	4.0	0.3	–	–	0.3	0.3	0.4	–
M2	0.1	0.2	0.1	–	–	–	–	–	–	–
M3	1.4	1.0	1.9	–	–	–	0.1	0.1	0.1	–
M4	18.5	21.4	12.9	–	–	–	7.2^c^	4.9^c^	5.0^c^	–
M5	30.1	16.4	11.9	–	–	–	2.0	0.5	0.4	–
M6	0.04	0.7	0.4	–	–	–	–	–	–	–
M7	0.3	4.9	3.7	0.2	–	–	0.1	0.1	0.1	0.5
M8	0.6	0.3	1.8	–	–	–	0.3^c^	0.3^c^	2.2^c^	–
M9	3.1	4.8	3.1	–	–	–	1.4^c^	0.5^c^	0.8^c^	–
M10	23.7	18.6	16.0	–	–	–	0.8	0.3	0.3	–
M11	0.3	1.3	2.2	–	–	–	–	–	–	–
M12	0.2	4.4	2.9	–	–	–	–	–	–	–
M13	1.2	0.4	2.6	–	–	–	–	–	–	–
M14	8.3	3.9	6.8	–	–	–	3.3^c^	1.2^c^	2.3^c^	–
M15	0.4	0.5	1.5	–	–	–	0.1^c^	0.5^c^	0.8^c^	–
M16	1.8	1.2	7.2	0.5	0.3	1.6	0.8	2.4	2.6	10.1
M17	1.2	0.3	1.8	0.1	–	–	1.1	3.2	1.9	–
M18	0.5	0.1	0.5	–	–	–	0.5^d^	0.5^d^	0.1^d^	–
M19	5.5	1.8	0.7	0.1	–	–	–	–	–	–
M20	0.2	1.2	0.1	–	–	–	–	–	–	–
M21	–	–	–	–	–	–	0.4^d^	0.5^d^	0.6^d^	–
M22	–	–	–	–	–	–	3.7	1.2	1.1	–
M23	–	–	–	–	–	–	2.9	0.9	0.1	–
M24	–	–	–	–	–	–	2.1	0.5	0.0	–
M25	–	–	–	–	–	–	4.5	9.8	5.1	7.8
M26	–	–	–	–	–	–	5.2	3.6	4.1	–
M27	–	–	–	–	–	–	1.2	4.1	1.5	0.2
M28	–	–	–	–	–	–	12.1	7.4	10.3	–
M29	–	–	–	–	–	–	2.0	8.5	2.6	0.2
M30	–	–	–	–	–	–	14.3	7.8	16.0	0.4
M31	–	–	–	–	–	–	3.0	10.4	4.4	0.2
M32	–	–	–	–	–	–	12.9	5.1	13.4	0.7
M33	–	–	–	–	–	–	2.9	9.0	4.9	0.4
M34	–	–	–	–	–	–	10.7	5.7	12.6	–
M35	–	–	–	–	–	–	3.8	6.3	4.5	0.5

^a^For liver S9 data, results were from 60 min in liver S9 and presented as percentages of the total peak area at 60 min time point from 10 µM incubation

^b^For hepatocyte data, results were from 120 min in hepatocytes and presented as percentages of the total peak area at 120 min time point from 10 µM incubation

^c^Data from 15 min time point

^d^Data from 60 min time point

In hepatocytes (across all species), 28 metabolites were detected (Table 5 and “Appendix 1” Table 15). All hepatocyte metabolites were detected in human, and the most abundant metabolites were O-dealkylation with 6 × hydroxylations and dehydrogenation (M28, 12.1%), O-dealkylation (loss of C_4_H_8_O_2_) with 6 × hydroxylations and dehydrogenation (M30, 14.3%), O-dealkylation (loss of C_6_H_12_O_3_) with 6 × hydroxylations and dehydrogenation (M32, 12.9%), and O-dealkylation (loss of C_8_H_16_O_4_) with 6 × hydroxylations and dehydrogenation (M34, 10.7%). In rat, the most abundant metabolites were M25 (9.8%) and M28–M35 (5.1–10.4%). In hamster, the most abundant metabolites were M28 (10.3%), M30 (16%), M32 (13.4%), and M34 (12.6%).

M2, M6, M11–M13, M19, and M20 were detected in liver S9 fraction but not in hepatocytes, while M21–M34 were detected in hepatocytes but not in liver S9 fraction.

### Metabolite identification C_16_EO_8_

The same 33 metabolites were detected for C_16_EO_8_ in liver S9 of all species (Table 6 and “Appendix 1” Table 16). The most abundant metabolites in all species were hexadecane di-hydroxylation and dehydrogenation M3 (3.8%), O-dehexadecylation M4 (4.8%), and O-dealkylation M12 (4.6%).

**Table 6:** Metabolite profiles for C_16_EO_8_

Metabolite code	Liver S9 with cofactors, %^a^			Liver S9 without cofactors, %^a^			Hepatocytes, %^b^			
	Human	Rat	Hamster	Human	Rat	Hamster	Human	Rat	Hamster	Buffer
C_16_EO_8_	72.0	85.8	82.7	96.6	98.7	97.4	30.3	1.0	10.6	29.7
M1	0.1	0.5	0.2	–	–	–	0.8	0.1	0.1	–
M2	0.2	0.1	0.1	–	–	–	–	–	–	–
M3	3.8	1.2	2.1	–	–	–	12.3	0.2	2.7	–
M4	4.8	4.8	1.0	0.9	0.3	0.5	15.9	84.4	45.9	13.7
M5	1.0	0.1	0.2	0.05	0.01	0.04	3.3	5.1	2.7	0.5
M6	0.7	0.06	0.2	0.04	0.01	0.03	1.1	0.6	0.5	0.6
M7	0.8	0.04	0.1	0.04	0.01	0.03	1.4	0.2	0.4	1.0
M8	0.5	0.2	0.2	0.30	0.11	0.6	0.7	0.3	0.4	36.5
M9	0.3	0.04	0.08	0.015	0.002	0.035	0.4	0.1	0.2	1.1
M10	0.2	0.04	0.07	0.01	–	0.02	0.2	0.04	0.1	0.4
M11	0.2	0.1	0.1	0.03	0.01	0.4	0.3	0.1	0.2	16.2
M12	4.6	2.5	3.7	1.8	0.3	0.5	–	–	–	–
M13	1.1	0.5	1.9	–	–	–	–	–	–	–
M14	0.3	0.3	1.9	0.1	0.1	0.2	–	–	–	–
M15	0.1	0.1	2.0	0.1	0.3	0.2	–	–	–	–
M16	0.06	0.1	0.5	0.06	0.06	0.07	–	–	–	–
M17	1.5	0.9	0.7	–	–	–	2.8	0.1	0.3	–
M18	0.9	0.7	0.6	–	–	–	–	–	–	–
M19	0.2	0.3	0.4	–	–	–	–	–	–	–
M20	0.06	0.1	0.3	–	–	–	–	–	–	–
M21	1.0	0.2	0.1	–	–	–	0.9	0.03	0.07	–
M22	0.9	0.3	0.05	–	–	–	0.2	0.02	0.02	–
M23	0.6	0.5	0.03	–	–	–	–	–	–	–
M24	0.3	0.3	0.05	–	–	–	–	–	–	–
M25	0.1	0.10	0.03	–	–	–	–	–	–	–
M26	0.2	0.05	0.05	–	–	–	–	–	–	–
M27	0.6	0.01	0.1	–	–	–	0.3	0.01	0.07	–
M28	1.1	0.03	0.1	–	–	–	0.3	0.01	0.04	–
M29	0.7	0.04	0.07	–	–	–	0.3	0.004	0.01	–
M30	0.5	0.03	0.04	–	–	–	–	–	–	–
M31	0.30	0.03	0.05	–	–	–	–	–	–	–
M32	0.09	0.04	0.05	–	–	–	–	–	–	–
M33	0.09	0.04	0.15	–	–	–	–	–	–	–
M34	–	–	–	–	–	–	4.7	0.2	0.4	–
M35	–	–	–	–	–	–	4.6	0.03	0.1	–
M36	–	–	–	–	–	–	3.4	0.1	0.1	–
M37	–	–	–	–	–	–	2.5	0.2	0.1	–
M38	–	–	–	–	–	–	2.1	0.2	0.1	–
M39	–	–	–	–	–	–	2.1	0.2	0.1	–
M40	–	–	–	–	–	–	6.1	3.1	24.1	–
M41	–	–	–	–	–	–	0.1	0.3	0.3	–
M42	–	–	–	–	–	–	0.3	0.1	1.5	–
M43	–	–	–	–	–	–	0.5	0.2	0.6	–
M44	–	–	–	–	–	–	1.0	0.8	0.7	0.2
M45	–	–	–	–	–	–	0.4	2.7	4.8	0.3
M46	–	–	–	–	–	–	0.1	0.1	0.4	–
M47	–	–	–	–	–	–	0.3	0.1	0.6	–
M48	–	–	–	–	–	–	0.0	0.2	1.2	–
M49	–	–	–	–	–	–	0.2	0.2	0.8	–

^a^For liver S9 data, results were from 60 min in liver S9 and presented as percentages of the total peak area at 60 min time point from 10 µM incubation

^b^For hepatocyte data, results were from 120 min in hepatocytes and presented as percentages of the total peak area at 120 min time point from 10 µM incubation

In hepatocytes, 32 metabolites were detected in all species for C_16_EO_8_ (Table 6 and “Appendix 1” Table 16). In human, M3 (12.3%) and M4 (15.9%) were most abundant, while in rat and hamster, M4 (84.4% in rat and 45.9% in hamster) was the most abundant.

M2, M12–M16, M18–M20, M23–M26, and M30–M33 were detected in liver S9 fraction, but not in hepatocytes while M34–M49 were detected in hepatocytes but not in liver S9 fraction.

### Metabolite identification for C_18_EO_3_

In liver S9 fraction, a total of 11 metabolites were detected for C_18_EO_3_, all of them were found in human liver S9 whereas in rat and hamster liver S9, not all metabolites could be detected (Table 7 and “Appendix 1” Table 17). The most abundant metabolites in human liver S9 were octadecane di-hydroxylation and dehydrogenation M5 (13.7%), and octadecane hydroxylation with glucuronide conjugation M9 (20.1%). In rat and hamster liver S9, octadecane hydroxylation M1 (8% in rat and 12.4% in hamster) was the most abundant metabolite, while in hamster liver S9, also M5 (5.3%) had relatively high abundance.

**Table 7:** Metabolite profiles for C_18_EO_3_

Metabolite code	Liver S9 with cofactors, %^a^			Liver S9 without cofactors, %^a^			Hepatocytes, %^b^			
	Human	Rat	Hamster	Human	Rat	Hamster	Human	Rat	Hamster	Buffer
C_18_EO_3_	58.2	90.3	79.9	100.0	100.0	100.0	8.9	98.3	97.5	–
M1	2.6	8.0	12.4	–	–	–	–	–	–	–
M2	0.5	–	–	–	–	–	–	–	–	–
M3	0.2	0.2	0.3	–	–	–	–	–	–	–
M4	0.1	0.4	0.1	–	–	–	–	–	–	–
M5	13.7	0.3	5.3	–	–	–	15.5	1.0	1.9	–
M6	1.2	0.04	0.6	–	–	–	–	–	–	–
M7	1.6	–	–	–	–	–	–	–	–	–
M8	0.5	0.5	0.3	–	–	–	–	–	–	–
M9	20.1	0.1	1.1	–	–	–	40.7	–	–	–
M10	1.1	–	–	–	–	–	–	–	–	–
M11	0.3	–	–	–	–	–	–	–	–	–
M12	–	–	–	–	–	–	1.1	–	–	–
M13	–	–	–	–	–	–	4.6	–	0.2	–
M14	–	–	–	–	–	–	3.1	–	–	–
M15	–	–	–	–	–	–	5.4	0.04	0.3	–
M16	–	–	–	–	–	–	5.7	0.2	–	–
M17	–	–	–	–	–	–	12.2	0.4	0.1	–
M18	–	–	–	–	–	–	2.9	–	–	–

^a^For liver S9 data, results were from 60 min in liver S9 and presented as percentages of the total peak area at 60 min time point from 10 µM incubation

^b^For hepatocyte data, results were from 120 min in hepatocytes and presented as percentages of the total peak area at 120 min time point from 10 µM incubation

In hepatocytes, 9 metabolites were detected in total for C_18_EO_3_ (Table 7 and “Appendix 1” Table 17). All 9 metabolites could be detected for human hepatocytes whereas for rat and hamster only 4 metabolites each could be measured. M9 (40.7%) and M5 (15.5%) were the main metabolites in human. In rat and hamster, detected metabolite levels were low, but M5 (1% in rat and 1.9% in hamster) appeared to also be the main metabolite in these species.

Only M5 and M9 were detected both in liver S9 and hepatocytes, while M12–M18 were detected only in hepatocytes.

### OECD QSAR toolbox prediction

The in silico metabolism simulation results with OECD QSAR Toolbox, including the predicted metabolites structures are presented in “Appendix 1” Table 18 and “Appendix 1” Tables 19, 20, 21, 22, 23, and a summary of the predicted metabolites and pathways is provided in Table 8.

**Table 8:** Metabolites and metabolic pathways identified from OECD QSAR toolbox

Hydrolysis (acidic)	
Metabolites	Pathway(s)/reaction(s
1. Alcohols	1. Hydrolysis of various ether groups
2. Alcohol mono and oligo ethylene glycol ethers. i.e. compounds where one or more EO units have been removed	
3. Mono and oligo ethylene glycols	

Hepatic metabolism (‘In vivo Rat’ and ‘Rat liver S9’)	
Metabolites	Pathway(s)/reaction(s
1. Alcohols, aldehydes and carboxylic acids	1. Hydrolysis of various ether groups
2. Fatty acids with hydroxyl and/or carbonyl group(s) inserted at various positions in the alkyl moiety	2. Oxidation of alcohols to aldehydes and fatty acids
3. Fatty acids with one or more removed C_2_-units	3. Oxidation of alkyl moieties by insertion of hydroxyl group(s) and their subsequent conversion to carbonyl group(s)
4. Alcohol mono and oligo ethylene glycol ethers i.e. compounds where one or more EO units have been removed	4. β-oxidation of fatty acids, i.e. removal of C_2_-units
5. Alcohol mono and oligo ethylene glycol ethers with hydroxyl and/or carbonyl group(s) inserted at various positions in the alkyl moiety	5. Oxidation of mono and oligo ethylene glycols, either as such after hydrolysis or bound to an alkyl moiety
6. Mono and oligo ethylene glycols	
7. Partially and fully oxidised compounds originating from mono and oligo ethylene glycols containing hydroxyl and/or carbonyl and/or acidic groups, e.g. glycolaldehyde, glycolic acid, glyoxylic acid and finally oxalic acid, 8-hydroxy-3,6-dioxaoctanal, β-hydroxyethoxyacetic acid and 2-carboxymethoxy-ethoxy)-acetic acid	
8. Alcohol mono and oligo ethylene glycol ethers where the ethylene glycol moiety is partially or fully oxidised, containing carbonyl or carboxyl (acid) groups	

Skin metabolism	
Metabolites	Pathway(s)/reaction(s
1. Aldehydes and fatty acids	1. Oxidation of alcohols to aldehydes and fatty acids
2. Alcohol mono and oligo ethylene glycol ethers where the glycolic hydroxyl group has been oxidised to a carbonyl group	2. Oxidation of glycolic hydroxyl groups

In general, all compounds considered were predicted to be metabolized following a common mechanism with the number of predicted metabolites increasing with increasing number of EO groups. From the metabolic conversions identified (“Appendix 1” Tables 19, 20, 21, 22, 23), hydrolysis of ether group will occur whenever possible and is predicted by the hydrolysis simulator as well as the hepatic simulators used. Both simulations demonstrated that all available ether groups are subject to hydrolysis, and hydrolysis can occur at various positions in the AEs investigated. In addition, alcohols originating from hydrolysis of the ether group between the alkyl chain and the EO groups are oxidized to carboxylic (fatty) acids via the intermediate stage of aldehydes. Fatty acids are degraded by β-oxidation (i.e. fatty acids containing 2, 4, 6, etc. C atoms less than the parent fatty acid or alcohol (removed C_2_-units)) are simulated. Furthermore, the insertion of hydroxyl groups at various positions in the alkyl chain and their subsequent oxidation to carbonyl groups (i.e. dehydrogenation) is simulated for all compounds. Moreover, available hydroxyl group(s) in mono and oligo ethylene glycol ethers are oxidized via the corresponding aldehyde(s) to acidic compounds, for example, glycolaldehyde, glycolic acid, glyoxylic acid, oxalic acid, 8-hydroxy-3,6-dioxaoctanal, β-hydroxyethoxyacetic acid and 2-carboxymethoxy-ethoxy)-acetic acid.

## Discussion

Metabolism is considered to be one of the most important factors impacting the potential of a chemical to cause toxicity (Nebbia 2012). The results of our present investigation with AEs sheds light on the metabolic profile and the mechanism of biotransformation of AEs in both liver S9 and hepatocytes from humans, rats and hamsters.

Metabolic stability and clearance were measured in vitro (in both liver S9 and hepatocytes) to study the kinetic properties of AEs across a variety of species (human, rat, and hamster). In general, all AEs were metabolized by liver S9 and hepatocytes from human, rat, and hamster. There were some analytical issues with the detection of the disappearance of AEs in both test system when the alkyl chain length of AEs was greater than C12. As these AE are more hydrophobic compared to the shorter C Chain homologues, the potential cause of this may be due to non-specific binding to the incubation wells which has been reported by other researchers (Proença et al. 2021). Additionally, some technical analytical limitations are possible. Due to the poor quality of the LC/MS data for the higher C Chain AE homologues, it was not possible to reliably calculate half-life/clearance (shown as NA in Table 2). Furthermore, some estimations are considered less accurate due to a substantial fluctuation of the LC/MS peak areas pertaining to C_16_EO_8_ and C_18_EO_3_, which may be due to their hydrophobic and poorly soluble nature with increased potential for binding to the plastic incubation well walls. Therefore, it could not be concluded which factors (i.e. alkyl chain or EO group) influence the metabolism rate for these compounds in both metabolizing systems.

Specifically, the *t*1/2 values obtained for the 1 µM and 10 µM incubations were comparable with both C8EO4 and C10EO5. Therefore, these AEs can be considered to be metabolized quickly and there are no significant variations between the two initial concentrations within each metabolizing system (liver S9 vs. hepatocytes).

Interestingly, we also observed differences between the two metabolizing systems, where AEs were metabolized faster in human than rat in liver S9, whereas the opposite was observed in the hepatocyte test systems. However, all of the AE compounds were metabolised within comparable timeframes. A reason for the small differences might be due to the well-known variances in cellular uptake of compounds and/or membrane permeability, which may contribute to species differences observed in the hepatocyte data. However, in some cases, this study suggests a more efficient metabolic breakdown in the presence of a human metabolic system (liver S9). In addition, the human kinetic in vitro data in this study shows good agreement with the observations detailed in the HERA report (HERA 2009) where 75% of radiolabeled C_12_EO_6_ and C_13_EO_6_ were excreted in human male volunteers within the first 24 h (Drotman 1980). The results are also consistent with prior observations of almost complete excretion with 24 h after exposure to C_16_EO_8_ (139 min with 1 µM in liver S9) which was the longest half-life established in this study.

Based on the metabolic profiles of liver S9 and hepatocytes, a potential metabolism pathway for each AE could be established since it was evident that there were filiations within the identified metabolites (see “Appendix 1” Table 13, 14, 15, 16, 17 and “Appendix 2” Figs. 5, 6, 7, 8, 9, 10, 11, 12, 13, 14). For example, in C_8_EO_4_ metabolism, the parent compound predominantly occurred with one or two hydroxyl groups (–OH) at the octane part (M1, M2, M3, M4, M5, and M6) probably via the omega or omega-1 oxidation route. This step of hydroxylation is most likely induced by cytochrome P450 (CYP450) enzymes (i.e. monooxygenases) which is frequently seen in hydrocarbon metabolism which inserts one molecular oxygen atom into the substrate (Miura 2013; Ortiz de Montellano 2010). Subsequently M1, M2, M3, or M4 is further oxidized to form the aldehyde (–CHO, M7, or M21) and subsequent carboxylic acid (–COOH, M9) at the octane terminal if it followed the omega oxidation route, or form the keto (R-CO-CH3, M7, or M21) if it followed the omega-1 oxidation route (Krettler et al. 2020; Miura 2013). Alternatively, the EO groups of the parent compound form carboxylic acid (–COOH) via hydroxylation and dehydrogenation (M8) which is observed in PEG metabolism and is mediated by alcohol dehydrogenase (ADH) and aldehyde dehydrogenase (ALDH) (Webster et al. 2007; Zakhari 2006). Subsequently, M8 is oxidized (via CYP450) from the PEG end to shorten the EO groups via O-dealkylation which is the loss of one EO group C_2_H_4_O (Miura 2013). The oxidation process is repeated and expected to remove all the EO groups from the AEs. In addition, omega-1 oxidation is expected to occur during AE metabolism as evidenced by the observation of metabolites, which have exactly the same mass and proposed reactions, as was seen with, M7 and M21 in C_8_EO_4_ metabolism. Interestingly, it has been reported that omega-1 oxidation is the preferred route of metabolism in hamster (Lhuguenot et al. 1988). The report observed this to be the case in C_8_EO_4_ metabolism and not for other AEs. It also reported that glucuronidation occurred throughout the entire metabolism process. For instance, the parent compound (i.e. C8EO4) could be conjugated with glucuronidation directly (M17), after hydroxylation (M20), and after each O-dealkylation (M18 and M19).

**Fig. 3:**
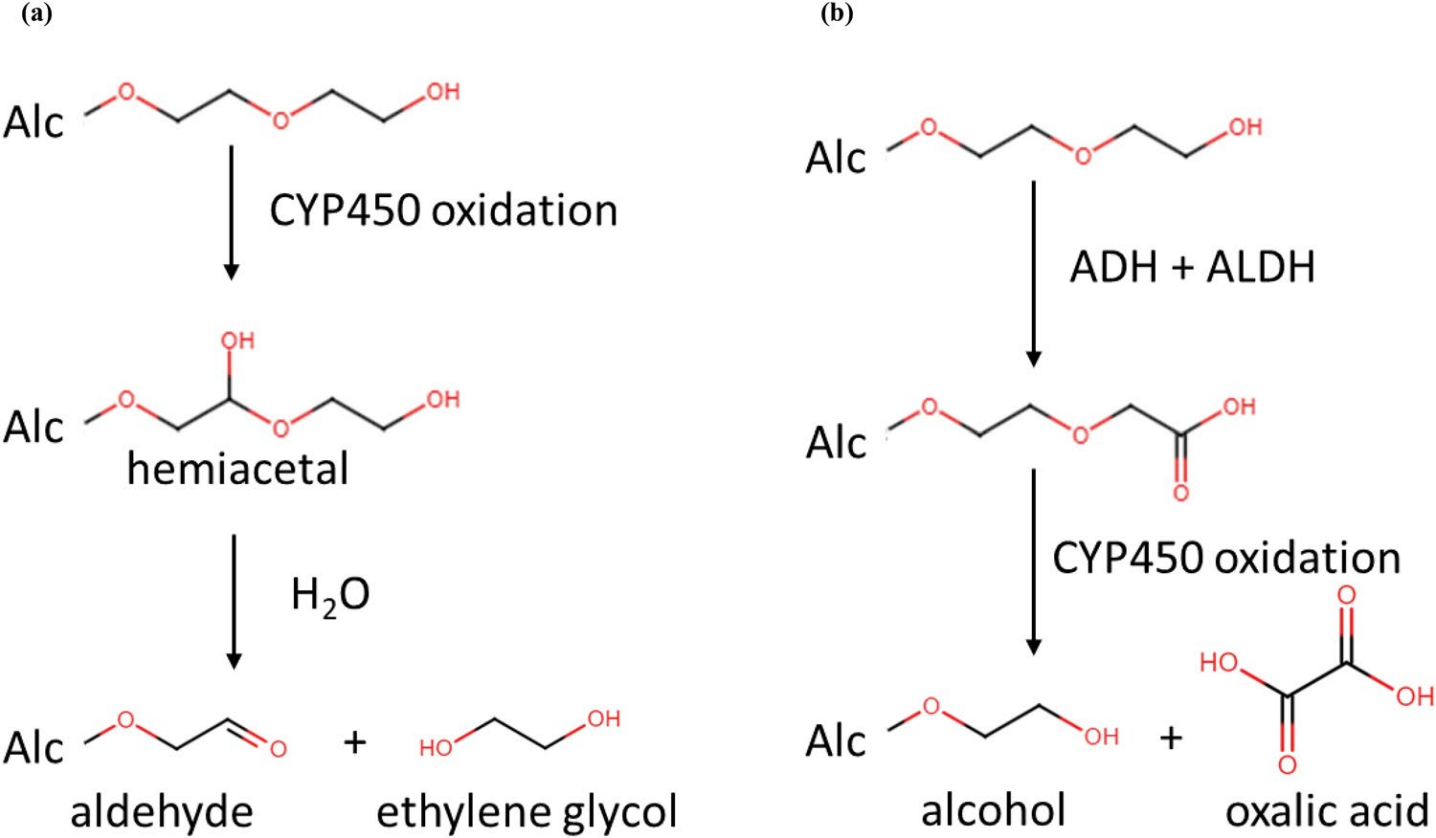
Potential shortening EO groups O-dealkylation process: **a** the EO groups of parent compound first oxidized to form a hemiacetal, then hydrolyzes to form a shorter EO groups with aldehyde terminal and a ethylene glycol; **b** the EO groups of parent compound first oxidized to form a carboxylic acid, then oxidizes to form a shorter EO groups with alcohol terminal and a oxalic acid

All in all, the metabolic profiles for human, rat, and hamster were compared to assess potential metabolic clearance pathways. For liver S9, the metabolite profiles of the three species were qualitatively similar although some quantitative differences were observed. In general, all investigated compounds were mono- and di-hydroxylations followed by dehydrogenation in the alkyl chain and further oxidation forming possibly carboxylic acids (–COOH). In addition, abundant metabolites via oxidation of EO groups, and shortening of EO groups (via consecutive losses of C_2_H_4_O) were detected in our study. For some of the compounds, low abundance glucuronide and sulfate conjugates were also observed after hydroxylation of the alkyl chain. Similar to liver S9, the metabolic profiles were qualitatively similar across all AEs although some of the minor metabolites were not detected in hepatocytes and several hepatocyte-specific metabolites were detected. Generally, the metabolism in hepatocytes seems to proceed further with several hydroxylation reactions and shortening of the EO groups, while the main metabolites detected in liver S9 fraction were most abundant in the earlier time points of hepatocyte experiments. Such phenomenon has previously been observed as this is the most significant difference between these two in vitro systems where hepatocytes contain the whole set of metabolic enzymes and cofactors at physiological levels (Li 2007). Interestingly, chain shortening was only observed at the site of the hydrophilic EO head groups, not at the hydrophobic alkyl chain. This may be due to the limitations of the analytical method in the current study and is not necessarily contradictory to metabolism of fatty alcohol cycle which has been reported previously (Rizzo 2014). Shortening of EO groups can be achieved by oxidative cleavage of C_2_H_4_O via CYP450 oxidative dealkylation (O-dealkylation) (Steber and Wierich 1985). During the shortening process, there are two possible oxidation processes based on our QSAR metabolites simulation (Appendix I Tables 19, 20, 21, 22, 23): 1) a hemiacetal maybe formed, then hydrolyzes to the shorter aldehyde plus ethylene glycol (Fig 3a), or 2) the hydroxyl group is oxidized to carboxylic acid, then further oxidized to the shorter alcohol plus oxalic acid (Fig 3b).  In addition, evidence from studies with PEG indicate that ethylene glycol is not formed as a metabolites of PEG in humans, but minor amounts of oxalic acid may be formed (Fruijtier-Pölloth 2005; Shaffer et al. 1950). However, small fragments, like ethylene glycol and oxalic acid, could not be detected in our current study due to technical limitations.

Cleavage of the ether bond between the alkyl chain and the EO groups was also detected in both liver S9 and hepatocytes (M15 in C_8_EO_4_, M25 in C_10_EO_5_, M16 in C_12_EO_4_, and M4 in C_16_EO_8_). Although there was no such metabolite (i.e. free 3 EO groups) identified in incubations with C_18_EO_3_, the existence of such a metabolite is highly probable. This can be explained by two facts: the existence of M17 which is a hydroxylated and dehydrogenated C18 alcohol, and the limitations of the current study. In addition, it is known that free alcohols and PEG are the by-products of AE biodegradation as the results of the “central fission” pathway, and such a metabolism pathway may also be applicable in mammalian cells (Swisher 1986; Szymanski et al. 2000). In principle, the 3EO metabolite should be detected since the percentage of whole EO groups increased with increasing alkyl chain where 0.6–1.5% for C_8_EO_4_, 2.0–3.2% for C_10_EO_5_, 0.8–2.4% for C_12_EO_4_, and 4.8–84.4% for C_16_EO_8_ was found. The analytical method was capable of detecting AEs with 3 or more EO units with good sensitivity, but this in fact did not occur. Additionally, fatty alcohols (without an EO head group) were not detected and the detection sensitivity for fatty alcohols with one EO group was extremely poor as these were not ionized in ESI or APCI ionization techniques. Similar technical difficulties have also been reported by other groups (Zembrzuska 2017).

**Fig. 4:**
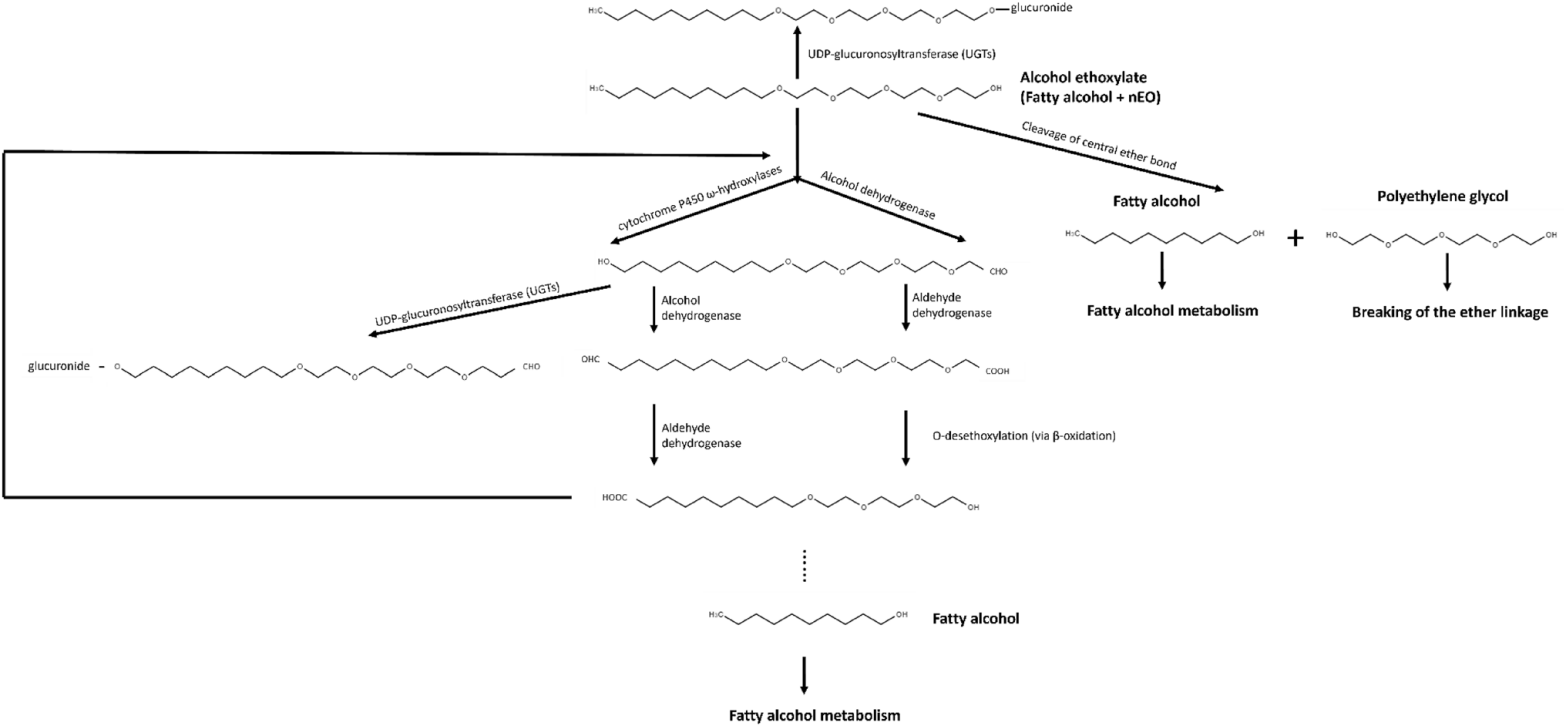
Proposed general AEs metabolism pathway identified in current study

**Fig. 5:**
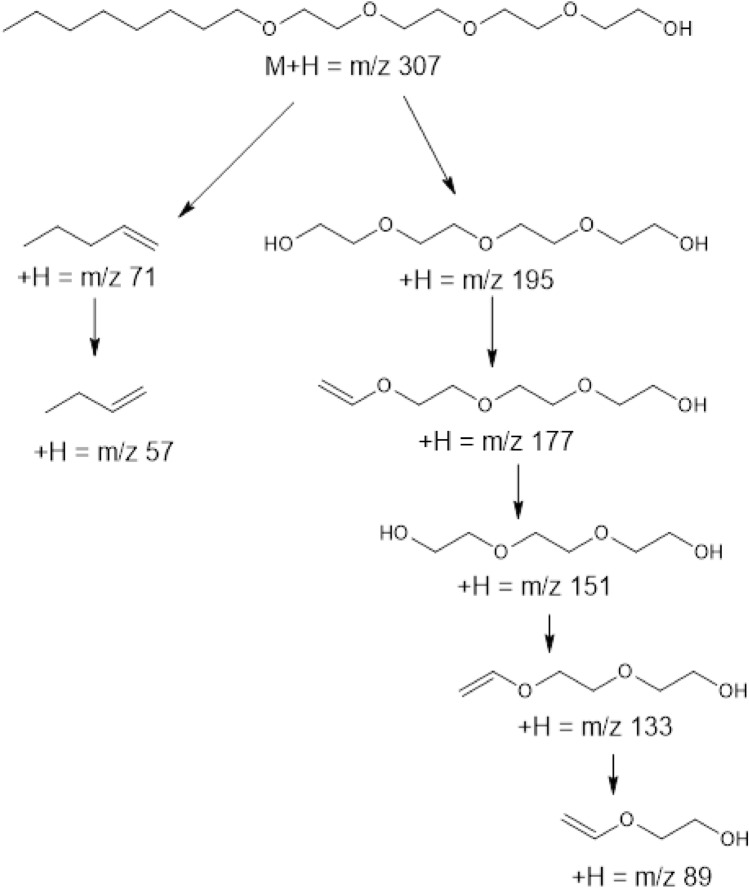
MS/MS fragment ion identification for C_8_EO_4_

**Fig. 6:**
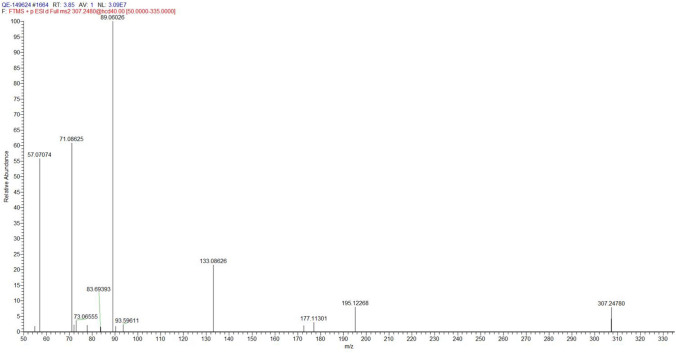
MS/MS spectrum of C_8_EO_4_

**Fig. 7:**
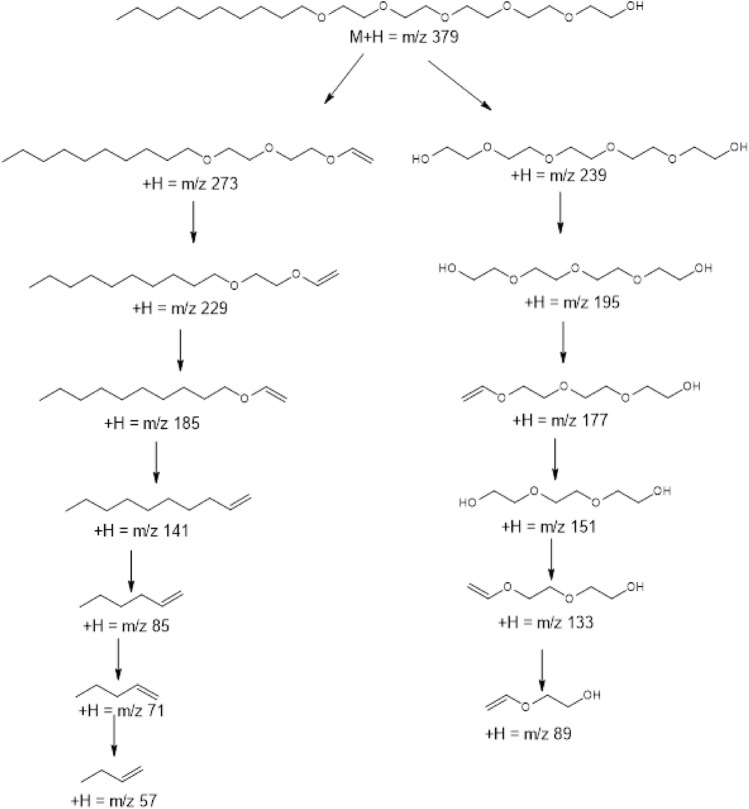
MS/MS fragment ion identification for C_10_EO_5_

**Fig. 8:**
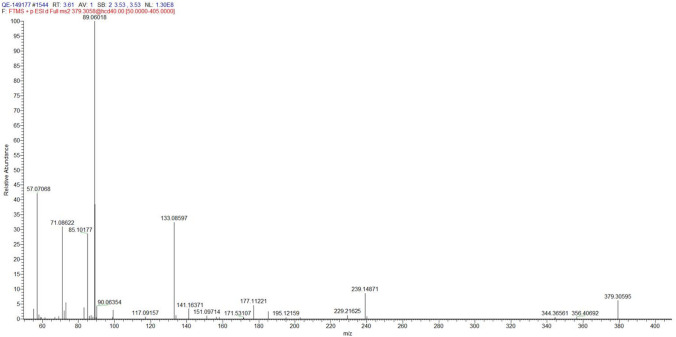
MS/MS spectrum of C_10_EO_5_

**Fig. 9:**
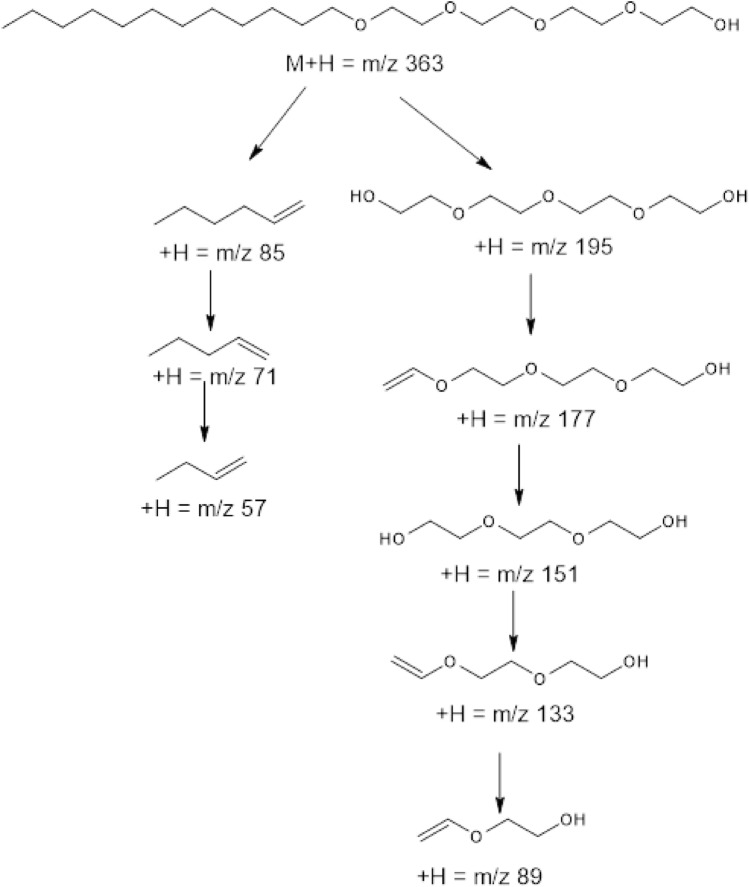
MS/MS fragment ion identification for C_12_EO_4_

**Fig. 10:**
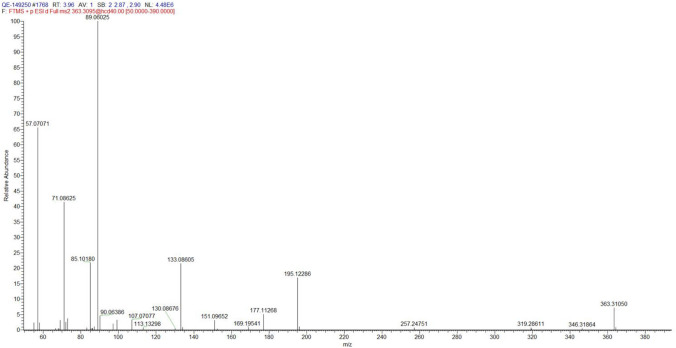
MS/MS spectrum of C_12_EO_4_

**Fig. 11:**
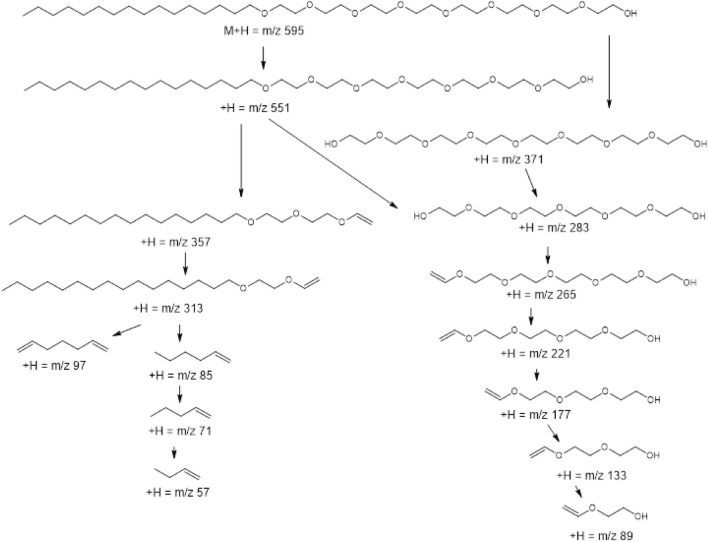
MS/MS fragment ion identification for C_16_EO_8_

**Fig. 12:**
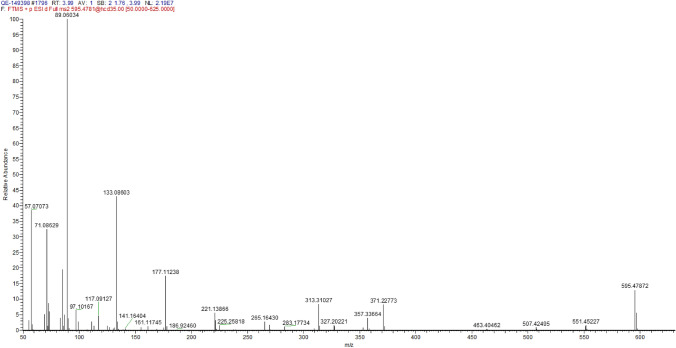
MS/MS spectrum of C_16_EO_8_

**Fig. 13:**
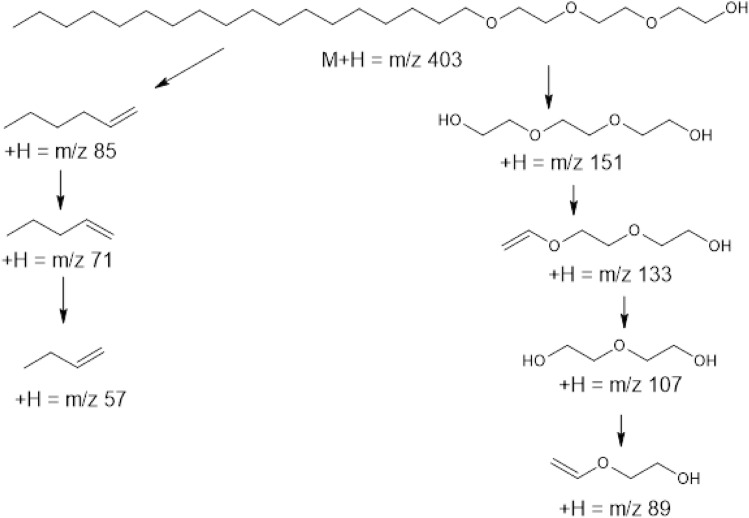
MS/MS fragment ion identification for C_18_EO_3_

**Fig. 14:**
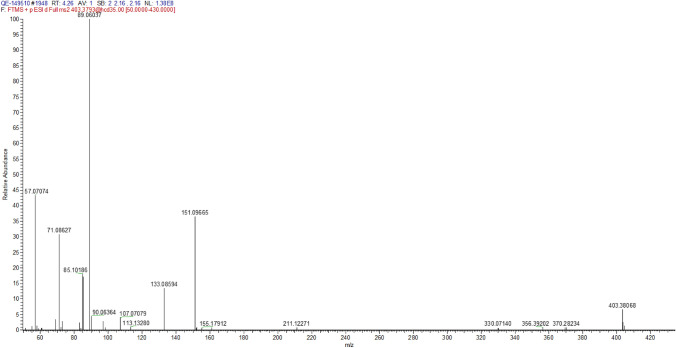
MS/MS spectrum of C_18_EO_3_

**Fig. 15:**
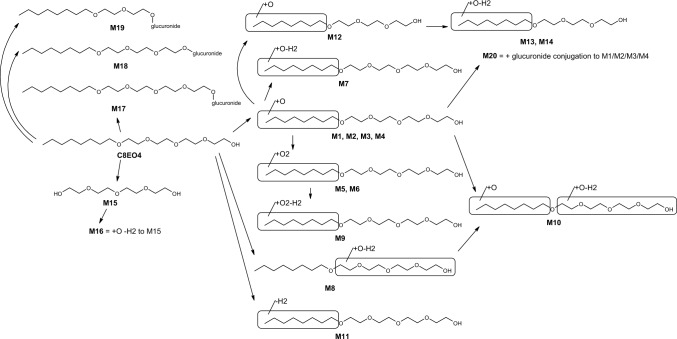
The suggested metabolic pathways for the observed C_8_EO_4_ metabolites in liver S9 fraction

**Fig. 16:**
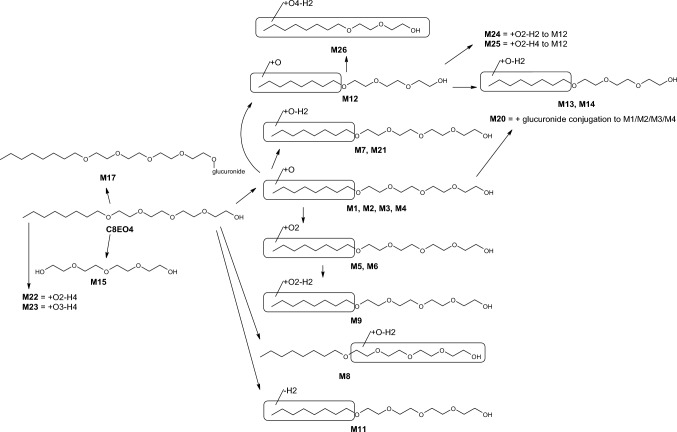
The suggested metabolic pathways for the observed C_8_EO_4_ metabolites in hepatocytes

**Fig. 17:**
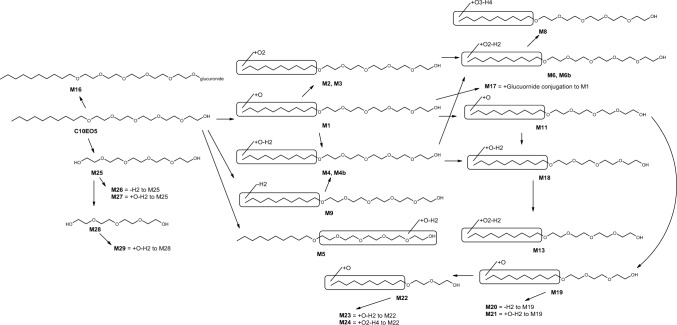
The suggested metabolic pathways for the observed C_10_EO_5_ metabolites in liver S9 fraction

**Fig. 18:**
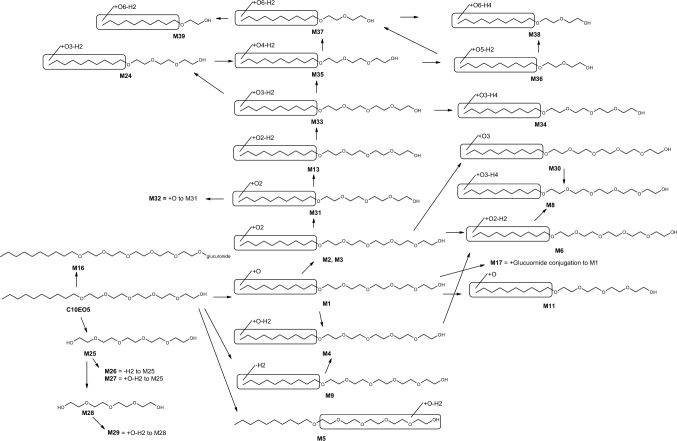
The suggested metabolic pathways for the observed C_10_EO_5_ metabolites in hepatocytes

**Fig. 19:**
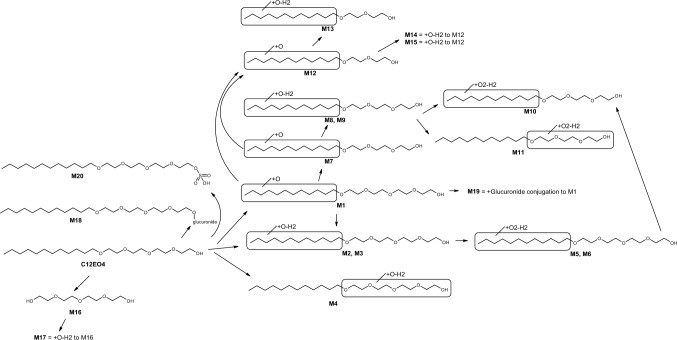
The suggested metabolic pathways for the observed C_12_EO_4_ metabolites in liver S9 fraction

**Fig. 20:**
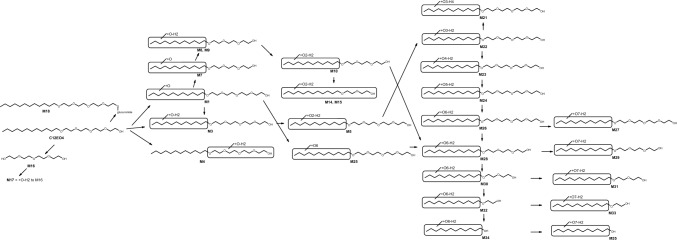
The suggested metabolic pathways for the observed C_12_EO_4_ metabolites in hepatocytes

**Fig. 21:**
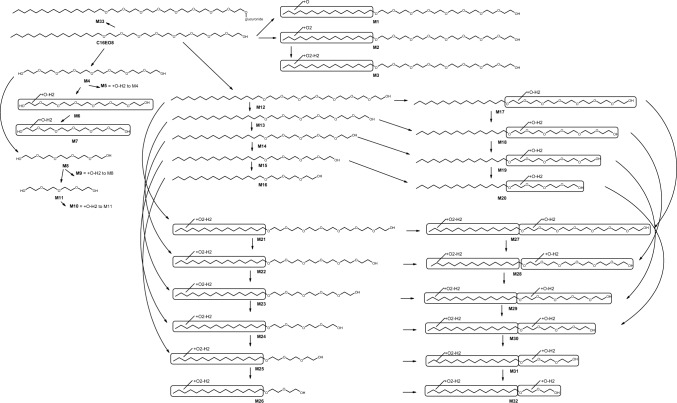
The suggested metabolic pathways for the observed C_16_EO_8_ metabolites in liver S9 fraction

**Fig. 22:**
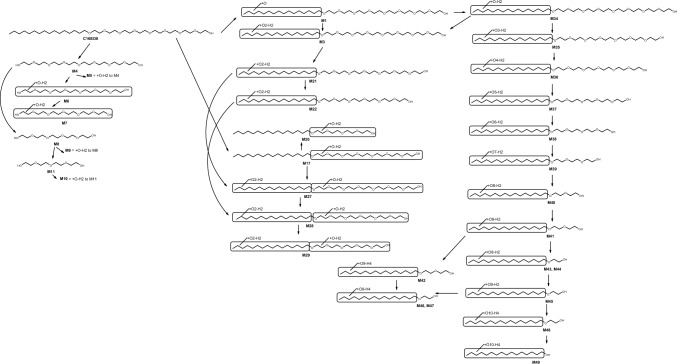
The suggested metabolic pathways for the observed C_16_EO_8_ metabolites in hepatocytes

**Fig. 23:**
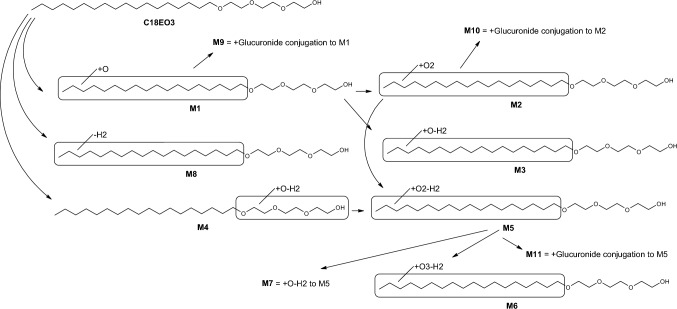
The suggested metabolic pathways for the observed C_18_EO_3_ metabolites in liver S9 fraction

**Fig. 24:**
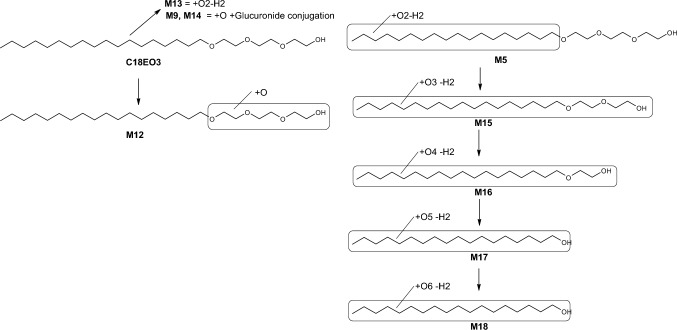
The suggested metabolic pathways for the observed C_18_EO_3_ metabolites in hepatocytes

In summary, based on the identified metabolites from each of the AEs, major similarities in metabolism were observed for the different AEs. Metabolism pathways for each AEs are shown in “Appendix 2” Figs. 15, 16, 17, 18, 19, 20, 21, 22, 23 and 24. Despite some of the analytical problems and limitations, the metabolites detected and identified allowed for general metabolism pathways to be derived (see Fig. 4). The in vitro data generated in this study provide a fresh insight into the metabolism of AEs and the results are aligned with HERA’s hypothesis that both the hydrophobic alkyl chain and the hydrophilic EO head groups are the main target sites for metabolism. Cleavage of the ether bond of AEs to form fatty alcohol and PEG is only a minor metabolism pathway within the in vitro test systems investigated in this study. It is recognized only ESI + was used in the current study, and it is suggested that for future investigations to the use of ESI− should be considered to enable some metabolites, such as sulfate conjugates, carboxylic acids, etc., to be detected and to add further to the complete picture of AE metabolism in vitro.

An excellent agreement between the in vitro experiments and the in silico metabolite predictions with OECD QSAR Toolbox was found. Both methodologies confirm that there is no difference in metabolic patterns of the various AEs evaluated, regardless of the alkyl chain length or the number of EO groups. None of the metabolites, either simulated or detected, indicates the presence different metabolic pathways or mechanisms. No unexpected chemical or enzymatic conversion resulting in the occurrence of unexpected metabolites was identified. When comparing the metabolite profiles in detail, not all metabolites observed in the in vitro experiments were simulated by the OECD QSAR toolbox and vice versa. No compounds with less than or equal to three EO groups and small fragments could be reliably detected using the LC–MS analytical method applied in this study, including *inter alia*, the non-ethoxylated alcohols and mono and oligo ethylene glycol derivatives, e.g. glycolaldehyde, glycolic acid, glyoxylic acid, and oxalic acid. These analytical limitations have been previously documented for alcohol ethoxylates in the scientific literature (Zembrzuska 2017). Furthermore, the OECD QSAR toolbox is also not capable of predicting phase II metabolism. These two explanations provide a reasonable basis to explain the differences between experimental in vitro and in silico metabolites in this study.

## Conclusions

This study provides substantial information on the metabolism of AEs in humans, rats, and hamster hepatic systems. The metabolic stability test of AEs in vitro indicates that all AEs have comparable metabolisms in liver microsomes, hepatocytes in all three species investigated. The metabolic rates of rodents (i.e. rats and hamsters) are similar to those of humans. All investigated AEs showed a similar metabolic pathway and metabolite profile across species. Although some quantitative differences were observed, indicating that the rat is likely to be an appropriate species for studies evaluating human health hazard endpoints for AEs. In both liver S9 and hepatocytes, metabolites were observed with hydroxylation (i.e. insertion of one oxygen in either the alkyl chain or EO groups or both), dehydrogenation (i.e. conversion of hydroxyl groups to carbonyl groups), O-dealkylation (i.e. via CYP450 oxidation to removal of C2 units from EO groups), glucuronidation proposed to be the major metabolic pathways. Cleavage of the ether bond is proposed to be a possible but minor metabolic pathway. Despite the minor differences identified for each of the individual homologue AE subject to testing, the authors assert that according to the EU RAAF guidance document, AEs from C8 to C18 can be grouped together based on their similarity of metabolism profile and metabolic rate.

## Figures and Tables

**Table 19:**
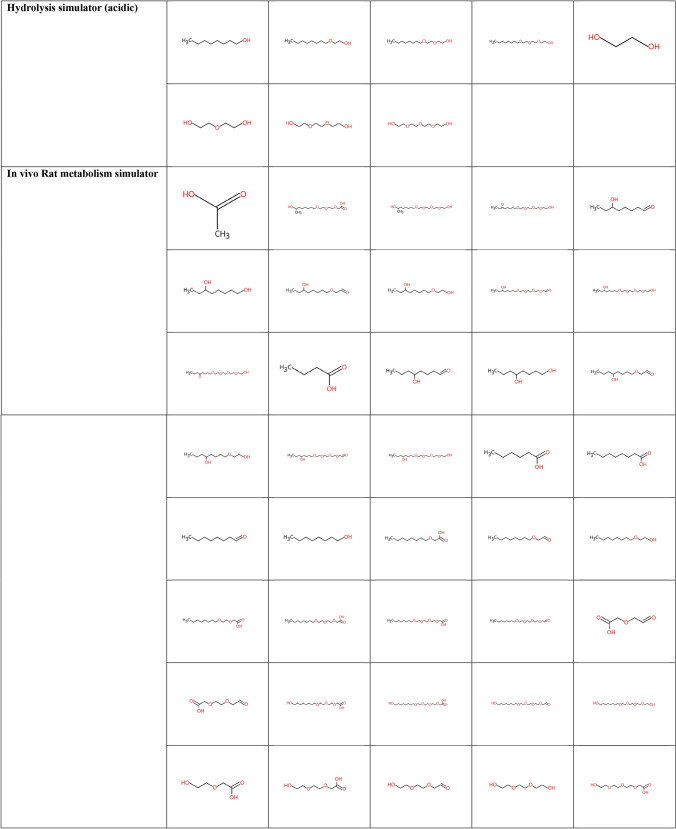

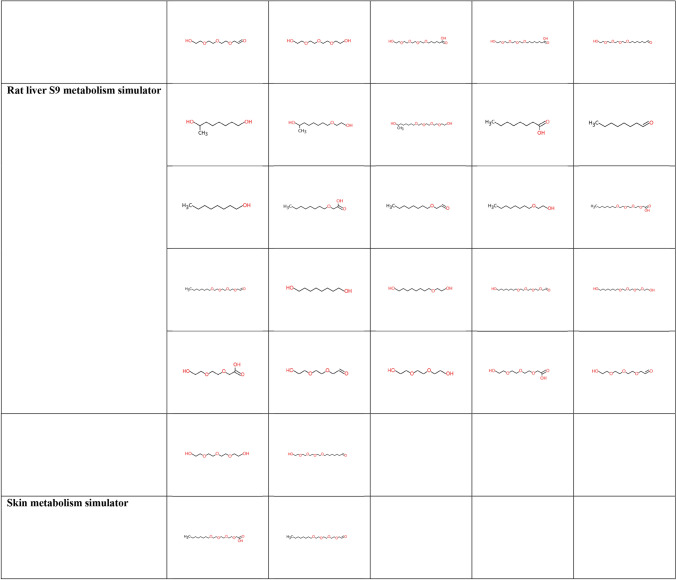
OECD QSAR toolbox metabolism profile simulation for C_8_EO_4_

**Table 20:**
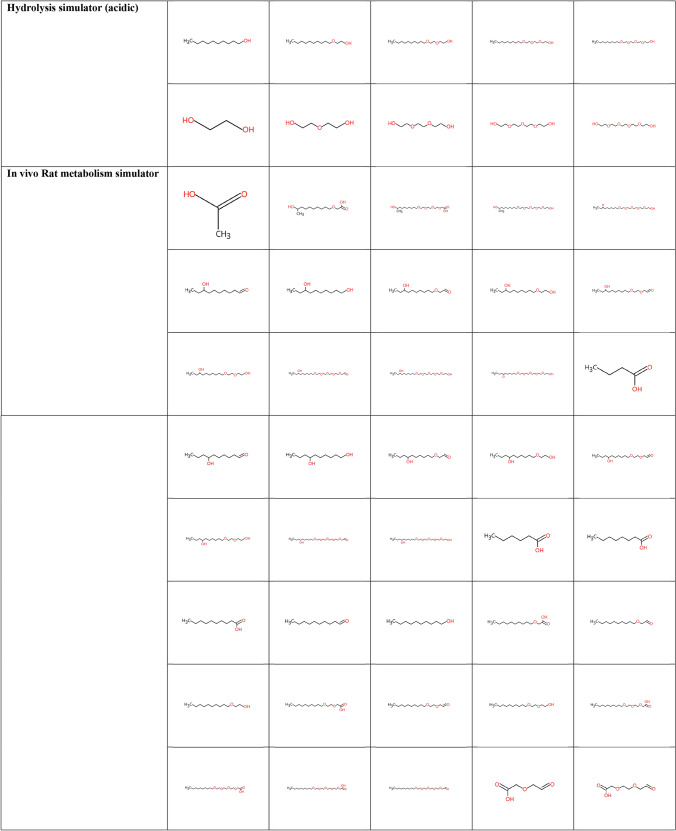

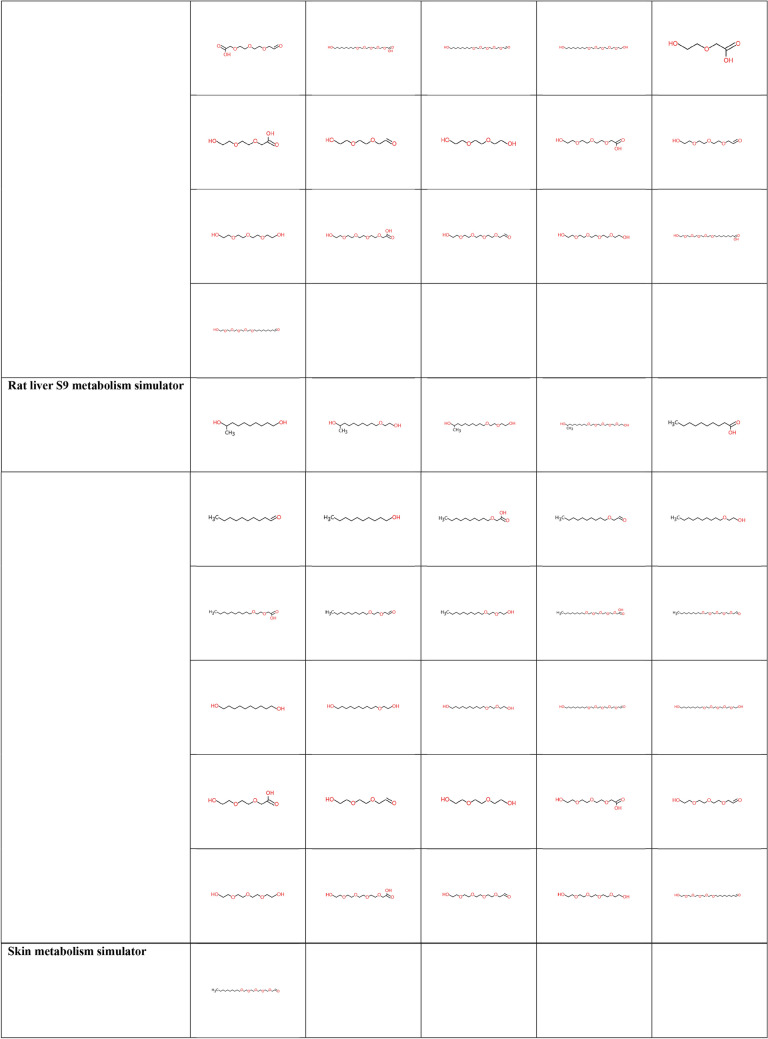
OECD QSAR toolbox metabolism profile simulation for C_10_EO_5_

**Table 21:**
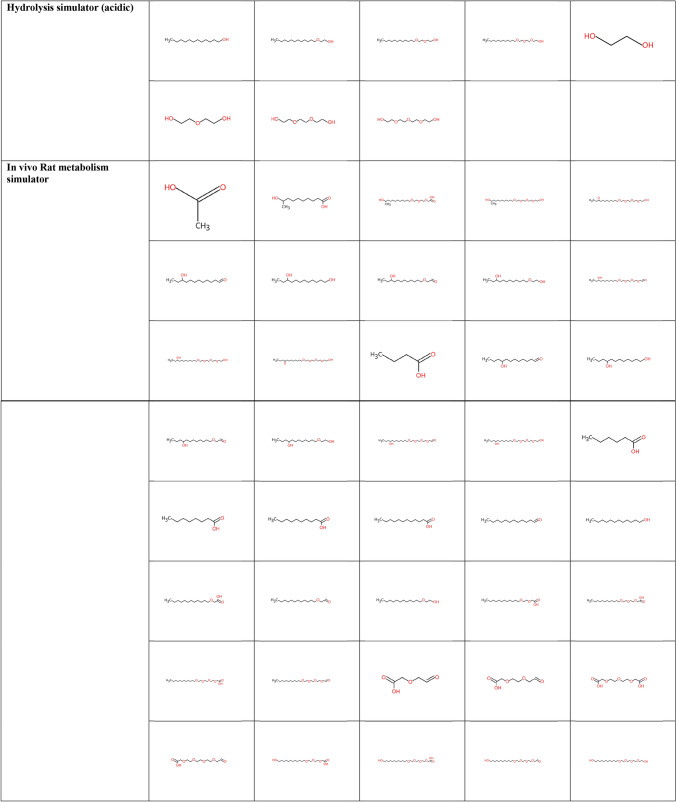

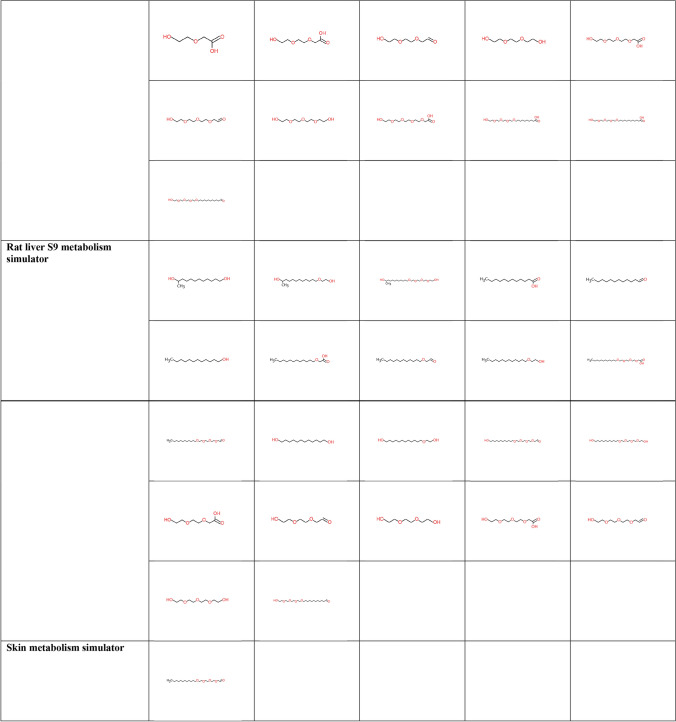
OECD QSAR toolbox metabolism profile simulation for C_12_EO_4_

**Table 22:**
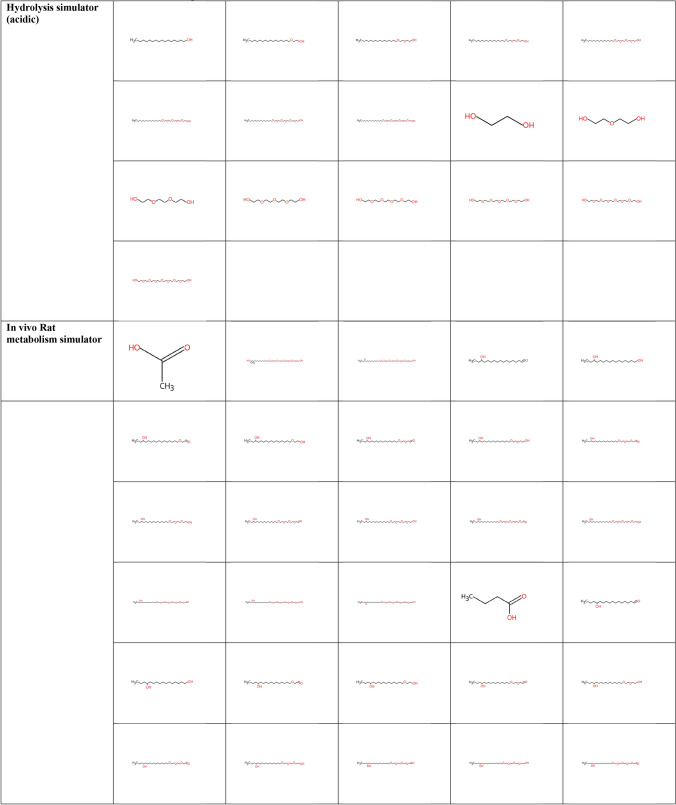

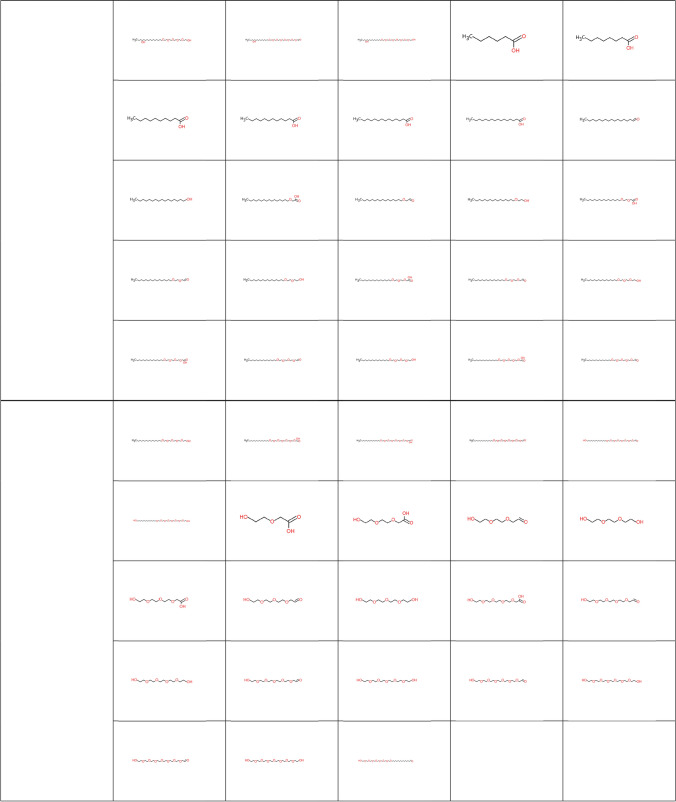

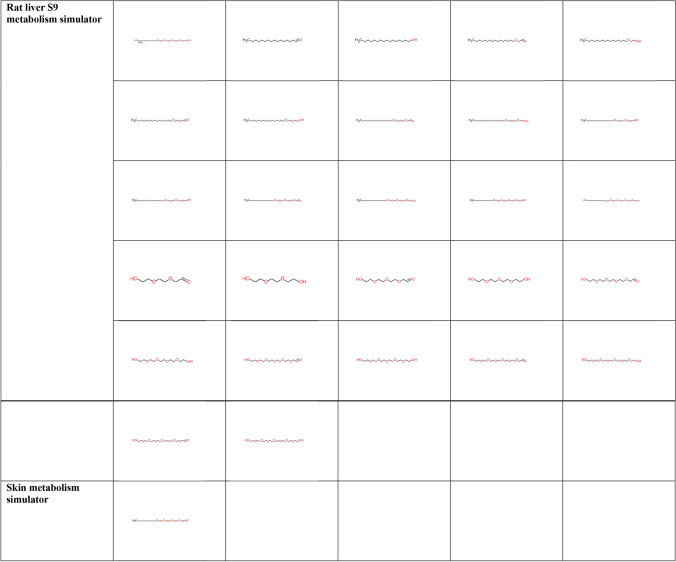
OECD QSAR toolbox metabolism profile simulation for C_16_EO_8_

**Table 23:**
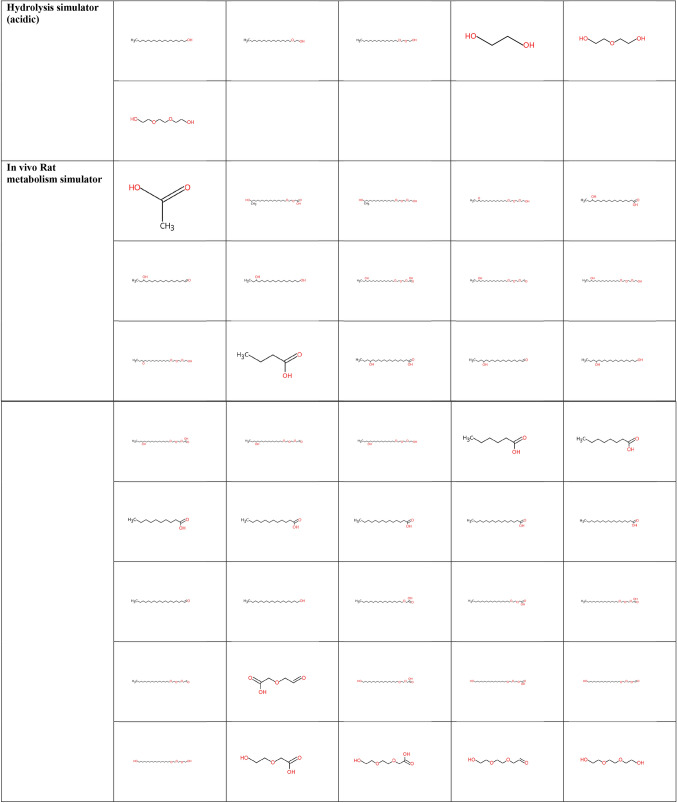

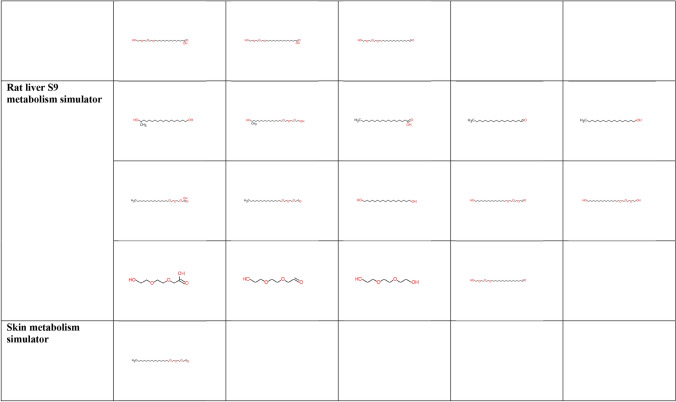
OECD QSAR toolbox metabolism profile simulation for C_18_EO_3_

## Data Availability

The data are available under the Archive of Toxicology. Any additional requests for data access should be directed to the corresponding author.

## Appendix 1

See Tables 9, 10, 11, 12, 13, 14, 15, 16, 17, 18, 19, 20, 21, 22 and 23.

**Table 9:** Characteristics of the used liver S9 fractions (activities by the supplier)

Mixed gender pooled human S9		
Celsis In Vitro Technologies product number X008023		
Lot number BKY		
Protein concentration	21.1 mg/ml	
Enzyme	Assay	Activity [jn pmol/(mg × min]
Total P450		0.099 nmol/mg
ECOD	TRoF 7-HC and metabolites	128
UGT	RoF 7-hydroxycoumarin glucuronide	676
CYP1A2	RoF acetaminophen	23.7
CYP2A6	RoF 7-HC and metabolites	60.8
CYP2B6	RoF hydroxybupropion	37.4
CYP2C8	RoF desethylamodiaquine	336
CYP2C9	RoF 4′-methylhydroxytolbutamide	62.2
CYP2C19	RoF 4′-hydroxymephenytoin	10.4
CYP2D6	RoF dextrorphan	10.6
CYP2E1	RoF 6-hydroxychlorzoxazone	141
CYP3A4	RoF 6β-hydroxytestosterone	296
CYP3A4	RoF 1-hydroxymidazolam	104

Male sprague dawley rat liver S9		
Celsis in vitro technologies product number M00002		
Lot number PQT		
Protein concentration	22.1 mg/ml	
Enzyme	Assay	Activity [jn pmol/(mg × min]
ECOD	TRoF 7-HC and metabolites	105

Male Syrian hamster liver S9		
Celsis in vitro technologies product number S00252		
Lot number MTL		
Protein concentration	20.7 mg/ml	
Enzyme	Assay	Activity [jn pmol/(mg × min]
ECOD	TRoF 7-HC and metabolites	677
UGT	7-OH-coumarin glucuronidation	1147
ST	7-OH-coumarin sulfatation	0

**Table 10:** Characteristics of the used hepatocytes (activities by the supplier)

Pooled cryopreserved human hepatocytes (50-donor mixed gender)		
Bioreclamation IVT product number X008005		
Lot number HQE		
Enzyme	Assay	Activity [jn pmol/10^6^ cells/min]
Total P450	ECOD	79.2
UGT	7-OH-coumarin glucuronidation	426
ST	7-OH-coumarin sulfatation	35.7
CYP1A2	Phenacetin O-deethylation	41.2
CYP2A6	Coumarin 7-hydroxylation	89.9
CYP2B6	Bupropion hydroxylation	35.6
CYP2C8	Amodiaquine de-ethylation	228
CYP2C9	Tolbutamide-4′-hydroxylation	52.0
CYP2C19	S-Mephenytoin 4′-hydroxylation	14.6
CYP2D6	Dextromethorphan demethylation	20.1
CYP2E1	Chlorzoxazone-6-hydroxylation	23.9
CYP3A4	Testosterone-6β-hydroxylation	137
CYP3A4	Midazolam-1-hydroxylation	69.4

Pooled cryopreserved male Sprague Dawley rat hepatocytes		
Bioreclamation IVT product number M00005		
Lot number PWX		
Enzyme	Assay	Activity [jn pmol/10^6^ cells/min]
Total P450	ECOD	122
UGT	7-OH-coumarin glucuronidation	163
ST	7-OH-coumarin sulfatation	97.7

Cryopreserved male Syrian hamster hepatocytes		
Bioreclamation IVT product number S001135		
Lot number GBI		
Enzyme	Assay	Activity [jn pmol/10^6^ cells/min]
Total P450	ECOD	501
UGT	7-OH-coumarin glucuronidation	271
ST	7-OH-coumarin sulfatation	40.8

**Table 11:** Stability data of control compound in Hepatocytes: Kinetic in vitro clearance and T_1/2_ for verapamil (1 µM). CL_int,inc,_ = intrinsic in vitro clearance, T_1/2_ = in vitro half-life

Species	µM	T_1/2_ (min)	CL_int,inc_ (µl/min/10^6^ cells)	Limit CL_int,inc_ (µl/min/10^6^ cells)
Human	1	22.7	30.6	> 15
Rat	1	< 11.4^a^	> 62^a^	> 58
Hamster	1	< 11.4^a^	> 62^a^	NA

^a^Complete disappearance in 120 min

**Table 12:** Stability data of control compounds in liver S9: Kinetic in vitro clearance and T_1/2_ for midazolam (1 µM). CL_int,inc,_ = intrinsic in vitro clearance, T_1/2_ = in vitro half-life

Species	µM	T_1/2_ (min)	CL_int,inc_ (µl/min/mg protein)	Limit T/12 (µl/min/mg protein)
Human	1	< 6.3^a^	> 75^a^	< 25
Rat	1	11.8	39.1	< 25
Hamster	1	< 6.3^a^	> 75^a^	NA

^a^Complete disappearance in 60 min

**Table 13:** Metabolite identification with UPLC/QE-orbitrap/MS data for C_8_EO_4_

Metabolite code	Retention time (min)	Calculated m/z	Proposed formula (M + H^+^)	Proposed reaction
C8EO4	3.86	307,2479	C16H35O5^+^	Parent compound
M1	2.57	323,2428	C16H35O6^+^	Hydroxylation in octane (Form a hydroxyl group at octane terminal or part)
M2	2.61	323,2428	C16H35O6^+^	Hydroxylation in octane (Form a hydroxyl group at octane terminal or part)
M3	2.68	323,2428	C16H35O6^+^	Hydroxylation in octane (Form a hydroxyl group at octane terminal or part)
M4	2.77	323,2428	C16H35O6^+^	Hydroxylation in octane (Form a hydroxyl group at octane terminal or part)
M5	2.05	339,2377	C16H35O7^+^	2 × Hydroxylation in octane (Form two hydroxyl groups at octane terminal or part)
M6	2.14	339,2377	C16H35O7^+^	2 × Hydroxylation in octane (Form two hydroxyl groups at octane terminal or part)
M7	2.59	321,2272	C16H33O6^+^	Hydroxylation + dehydrogenation in octane (probably omega or omega-1 oxidation of the hydroxyl group (i.e. M1, M2, M3 or M4) to an aldehyde at octane terminal or keto at octane part)
M8	3.91	321,2272	C16H33O6^+^	Hydroxylation + dehydrogenation in ethoxy (probably form carboxylic acid at ethoxy terminal)
M9	2.58	337,2221	C16H33O7^+^	2 × Hydroxylation + dehydrogenation in octane (probably omega oxidation, form carboxylic acid at octane terminal)
M10	2.68	337,2221	C16H33O7^+^	Hydroxylation + dehydrogenation in ethoxy + hydroxylation in octane (probably form carboxylic acid at ethoxy terminal and hydroxyl group at octane terminal)
M11	3.56	305,2323	C16H33O5^+^	Dehydrogenation in octane (Loss of 2 H form a double bond at octane part)
M12	2.54	279,2166	C14H31O5^+^	O-dealkylation + hydroxylation in octane (Loss of one EO unit (i.e.C2H4O) from the EO groups and form of hydroxyl group at octane terminal)
M13	2.51	277,2010	C14H29O5^+^	O-dealkylation + oxidation + dehydrogenation in octane (Loss of one EO unit (i.e.C2H4O) from the EO groups of M7 or M21)
M14	3.89	277,2010	C14H29O5^+^	O-dealkylation + oxidation + dehydrogenation in octane (Loss of one EO unit (i.e.C2H4O) from the EO groups of M7 or M21)
M15	1.30	195,1227	C8H19O5^+^	O-deoctylation (Loss of the entire octane)
M16	1.29	209,1020	C8H17O6^+^	O-deoctylation + hydroxylation + dehydrogenation (probably formation of carboxylic acid at ethoxy terminal of M15)
M17	3.47	500,3065	C22H46NO11	Glucuronide conjugation (NH3 adduct) (Glucuronidation of parent compound)
M18	3.41	456,2803	C20H42NO10	O-dealkylation + glucuronide conjugation (NH3 adduct) (Loss of one EO unit (i.e. C2H4O) from the EO groups of parent compound and then glucuronided)
M19	3.33	417,2095	C18H35NO9Na	O-dealkylation (loss of C4H8O2) + glucuronide conjugation (Na adduct) (Loss of two EO units (i.e. C4H8O2) from the EO groups of parent compound and then glucuronided)
M20	2.37	499,2749	C22H43O12	Hydroxylation + glucuronide conjugation (Glucuronidation of M1, M2, M3 or M4)
M21	2.64	321,2272	C16H33O6	Hydroxylation + dehydrogenation in octane (probably omega or omega-1 oxidation of the hydroxyl group (i.e. M1, M2, M3 or M4) to an aldehyde at octane terminal or keto at octane part)
M22	2.64	335,2064	C16H31O7	2 × Hydroxylation + 2 × dehydrogenation (probably form aldehyde at octane terminal and carboxylic acid at ethoxy terminal)
M23	2.62	351,2013	C16H31O8	3 × Hydroxylation + 2 × dehydrogenation (probably form carboxylic acid at both octane and ethoxy terminal)
M24	2.10	309,1908	C14H29O7	O-dealkylation + 3 × Hydroxylation + dehydrogenation (Loss of C2H4O from the EO groups, omega oxidation forming carboxylic acid at octane terminal, and form hydroxyl group at ethoxy terminal)
M25	2.56	307,1751	C14H27O7	O-dealkylation + 3 × Hydroxylation + 2 × dehydrogenation (Loss of C2H4O from the EO groups, omega oxidation forming carboxylic acid at octane terminal, and form carboxylic acid at ethoxy terminal)
M26	1.73	281,1595	C12H25O7	O-dealkylation (loss of C4H8O2) + 4 × Hydroxylation + dehydrogenation (Loss of two EO units (i.e. C4H8O2) from the EO groups, omega oxidation forming carboxylic acid at octane terminal, and form hydroxyl group at octane part or ethoxy terminal or both)

**Table 14:** Metabolite identification with UPLC/QE-orbitrap/MS data for C_10_EO_5_

Metabolite code	Retention time (min)	Calculated m/z	Proposed formula (M + H^+^)	Proposed reaction
C10EO5	3.61	379,3054	C20H43O6	Parent compound
M1	2.65	395,3003	C20H43O7	Hydroxylation in decane (Form hydroxyl group at decane terminal)
M2	2.04	411,2952	C20H43O8	2 × Hydroxylation in decane (form two hydroxyl groups at decane terminal or part)
M3	2.25	411,2952	C20H43O8	2 × Hydroxylation in decane (form two hydroxyl groups at decane terminal or part)
M4	2.67	393,2847	C20H41O7	Hydroxylation + dehydrogenation in decane (probably form aldehyde at decane terminal)
M4b	2.75	393,2847	C20H41O7	Oxidation + dehydrogenation in decane (probably form keto at decane part via omega-1 oxidation)
M5	3.63	393,2847	C20H41O7	Hydroxylation + dehydrogenation in ethoxy (probably form carboxylic acid at ethoxy terminal)
M6	2.61	409,2796	C20H41O8	2 × Hydroxylation + dehydrogenation in decane (probably omega oxidation form carboxylic acid at decane terminal or omega-1 oxidation form keto and hydroxyl group at decane part)
M6b	2.69	409,2796	C20H41O8	2 × Hydroxylation + dehydrogenation in decane (probably omega oxidation form carboxylic acid at decane terminal or omega-1 oxidation form keto and hydroxyl group at decane part)
M8	2.64	423,2589	C20H39O9	3 × Oxidation + 2 × dehydrogenation in decane (probably form a hydroxyl group and two keto groups at decane part)
M9	3.40	377,2898	C20H41O6	Dehydrogenation in decane (Loss of 2 H form a double bond at decane part)
M11	2.64	351,2741	C18H39O6	O-dealkylation + hydroxylation in decane (Loss of one EO unit (i.e. C2H4O) from the EO groups and form hydroxyl group at decane terminal or part)
M13	2.59	365,2534	C18H37O7	O-dealkylation + 2 × oxidation + dehydrogenation in decane (Loss of one EO unit (i.e. C2H4O) from the EO groups and form a keto and hydroxyl group at decane part)
M16	3.28	572,3641	C26H54NO12	Glucuronide conjugation (Glucuronidation of parent compound)
M17	2.37	571,3324	C26H51O13	Hydroxylation in decane + glucuronide conjugation (Glucuronidation of M1)
M18	2.64	366,2850	C18H40NO6	O-dealkylation + oxidation + dehydrogenation (NH3 adduct) (Glucuronidation of M4b)
M19	2.60	307,2479	C16H35O5	O-dealkylation (loss of C4H8O2) + oxidation in decane (Loss of two EO units (i.e. C4H8O2) from the EO groups, and form hydroxyl group at decane part)
M20	3.63	305,2323	C16H33O5	O-dealkylation (loss of C4H8O2) + oxidation + dehydrogenation (Loss of two EO units (i.e. C4H8O2) from the EO groups and form a keto (via omega-1 oxidation) at decane part)
M21	2.55	321,2272	C16H33O6	O-dealkylation (loss of C4H8O2) + 2 × oxidation + dehydrogenation (Loss of two EO units (i.e. C4H8O2) from the EO groups, and form a keto (via omega-1 oxidation) and form a hydroxyl group at decane part)
M22	2.57	263,2217	C14H31O4	O-dealkylation (loss of C6H12O3) + oxidation in decane (Loss of three EO units (i.e. C6H12O3) from the EO groups, and form hydroxyl group at decane part)
M23	2.52	277,2010	C14H29O5	O-dealkylation (loss of C6H12O3) + 2 × oxidation + dehydrogenation (Loss of three EO units (i.e. C6H12O3) from the EO groups, and form a keto (via omega-1 oxidation) and form a hydroxyl group at decane part)
M24	2.57	291,1802	C14H27O6	O-dealkylation (loss of C6H12O3) + 3 × oxidation + 2 × dehydrogenation (Loss of three EO units (i.e. C6H12O3) from the EO groups, and form two keto (via omega-1 oxidation) and form a hydroxyl group at decane part)
M25	1.37	239,1489	C10H23O6	O-decylation (loss of C10H20) (Loss of entire decane)
M26	1.28	237,1333	C10H21O6	O-decylation (loss of C10H20) + dehydrogenation (Loss of entire decane and form aldehyde group at ethoxy terminal)
M27	1.38	253,1282	C10H21O7	O-decylation (loss of C10H20) + oxidation + dehydrogenation (Oxidized aldehyde group of M26 to carboxylic acid)
M28	1.24	195,1227	C8H19O5	O-decylation (loss of C10H20) + O-dealkylation (Loss of one EO unit (i.e. C2H4O) of M25)
M29	1.23	209,1020	C8H17O6	O-decylation (loss of C10H20) + O-dealkylation + oxidation + dehydrogenation (Form carboxylic acid from M28)
M30	2.04	425,2745	C20H41O9	3 × Hydroxylation + dehydrogenation in decane (Form a carboxylic acid at decane terminal via omega oxidation and form a hydroxyl group at decane part)
M31	2.31	367,2690	C18H39O7	O-dealkylation + 2 × hydroxylation in decane (Loss of one EO unit (i.e. C2H4O) from the EO groups, and form two hydroxyl groups at decane terminal or part)
M32	2.74	365,2534	C18H37O7	O-dealkylation + 2 × hydroxylation + dehydrogenation (Loss of one EO unit (i.e. C2H4O) from the EO groups, and form one aldehyde group at decane terminal and a hydroxyl group at decane part or carboxylic acid group at decane terminal)
M33	2.28	381,2483	C18H37O8	O-dealkylation + 3 × hydroxylation + dehydrogenation in decane (Form one extra hydroxyl group at decane part of M32)
M34	2.65	379,2326	C18H35O8	O-dealkylation + 3 × hydroxylation + 2 × dehydrogenation in decane (Form keto from dehydrogenation of the hydroxyl group of M33)
M35	1.93	353,2170	C16H33O8	O-dealkylation (loss of C4H8O2) + 4 × hydroxylation + dehydrogenation in decane (Loss of two EO units (i.e. C4H8O2) from the EO groups, and form one aldehyde group at decane terminal and three hydroxyl groups at decane part or a carboxylic acid group at decane terminal and two hydroxyl groups at decane part)
M36	1.67	325,1857	C14H29O8	O-dealkylation (loss of C6H12O3 + 5 × hydroxylation + dehydrogenation in decane (Loss of three EO units (i.e. C6H12O3) from the EO groups, and form one aldehyde group at decane terminal and four hydroxyl groups at decane part or a carboxylic acid group at decane terminal and three hydroxyl groups at decane part)
M37	1.50	341,1806	C14H29O9	O-dealkylation (loss of C6H12O3 + 6 × hydroxylation + dehydrogenation in decane (Form one extra hydroxyl group at decane part of M36)
M38	1.69	339,1650	C14H27O9	O-dealkylation (loss of C6H12O3 + 6 × hydroxylation + 2 × dehydrogenation in decane (Form keto from dehydrogenation of the hydroxyl group of M37)
M39	1.48	297,1539	C12H25O8	O-dealkylation (loss of C8H16O4 + 6 × hydroxylation + dehydrogenation in decane (Loss of four EO units (i.e. C8H16O4) from the EO groups, and form one aldehyde group at decane terminal and five hydroxyl groups at decane part or a carboxylic acid group at decane terminal and four hydroxyl groups at decane part)

**Table 15:** Metabolite identification with UPLC/QE-orbitrap/MS data for C_12_EO_4_

Metabolite code	Retention time (min)	Calculated m/z	Proposed formula (M + H^+^)	Proposed reaction
C12EO4	3.98	363,3105	C20H43O5	Parent compound
M1	3.00	379,3054	C20H43O6	Hydroxylation in dodecane (Form hydroxyl group at dodecane terminal)
M2	2.29	377,2898	C20H41O6	Hydroxylation + dehydrogenation in dodecane (probably form aldehyde (via omega oxidation) at dodecane terminal or keto (via omega-1 oxidation) at dodecane part)
M3	3.04	377,2898	C20H41O6	Hydroxylation + dehydrogenation in dodecane (probably form aldehyde (via omega oxidation) at dodecane terminal or keto (via omega-1 oxidation) at dodecane part)
M4	3.27	377,2898	C20H41O6	Hydroxylation + dehydrogenation in ethoxy (probably form carboxylic acid at ethoxy terminal)
M5	2.94	393,2847	C20H41O7	2 × Hydroxylation + dehydrogenation in dodecane (probably form carboxylic acid at dodecane terminal of M2 or M3, or form one hydroxyl group and a keto at dodecane part)
M6	3.04	393,2847	C20H41O7	2 × Hydroxylation + dehydrogenation in dodecane (probably form carboxylic acid at dodecane terminal of M2 or M3, or form one hydroxyl group and a keto at dodecane part)
M7	2.98	335,2792	C18H39O5	O-dealkylation + hydroxylation in dodecane (Loss of one EO unit (i.e. C2H4O) from the EO groups, and form hydroxyl group at dodecane terminal)
M8	3.02	333,2636	C18H37O5	O-dealkylation + hydroxylation + dehydrogenation in dodecane (Loss of one EO unit (i.e. C2H4O) from the EO groups and form aldehyde at dodecane terminal or form keto at dodecane part)
M9	3.25	333,2636	C18H37O5	O-dealkylation + hydroxylation + dehydrogenation (Loss of one EO unit (i.e. C2H4O) from the EO groups and form aldehyde at dodecane terminal or form keto at dodecane part)
M10	2.92	349,2585	C18H37O6	O-dealkylation + 2 × hydroxylation + dehydrogenation in dodecane (Loss of one EO unit (i.e. C2H4O) from the EO groups form carboxylic acid at dodecane terminal, or form one hydroxyl group and a keto at dodecane part)
M11	3.03	349,2585	C18H37O6	O-dealkylation + 2 × hydroxylation + dehydrogenation in ethoxy (Loss of one EO unit (i.e. C2H4O) from the EO groups, form carboxylic acid at ethoxy terminal and form hydroxyl group at ethoxyl part)
M12	2.96	291,2530	C16H35O4	O-dealkylation (loss of C4H8O2) + hydroxylation in dodecane (Loss of two EO units (i.e. C4H8O2) from the EO groups and form hydroxyl group at dodecane terminal)
M13	3.00	289,2373	C16H33O4	O-dealkylation (loss of C4H8O2) + oxidation + dehydrogenation in dodecane (Loss of two EO units (i.e. C4H8O2) from the EO groups and form aldehyde at dodecane terminal or keto at dodecane part)
M14	2.90	305,2323	C16H33O5	O-dealkylation (loss of C4H8O2´) + 2 × oxidation + dehydrogenation (Loss of two EO units (i.e. C4H8O2) from the EO groups form carboxylic acid at dodecane terminal, or form one hydroxyl group and a keto at dodecane part)
M15	3.01	305,2323	C16H33O5	O-dealkylation (loss of C4H8O2) + 2 × oxidation + dehydrogenation (Loss of two EO units (i.e. C4H8O2) from the EO groups form carboxylic acid at dodecane terminal, or form one hydroxyl group and a keto at dodecane part)
M16	1.24	195,1227	C8H19O5	O-dodecylation (Loss of entire dodecane (C12H24))
M17	1.23	209,1020	C8H17O6	O-dodecylation + oxidation + dehydrogenation (Form carboxylic acid at ethoxy terminal of M16)
M18	2.91	556,3691	C26H54NO11	Glucuronide conjugation (NH3 adduct) (Glucuronidation of parent compound)
M19	2.64	555,3375	C26H51O12	Hydroxylation in dodecane + glucuronide conjugation (Glucuronidation of M1)
M20	2.80	459,2622	C20H43O9S	Hydroxylation in dodecane + sulfo-conjugation (Sulfation of M1)
M21	2.99	407,2639	C20H39O8	3 × Hydroxylation + 2 × dehydrogenation in dodecane (probably form keto from the hydroxyl group at dodecane part of M22)
M22	2.63	409,2796	C20H41O8	3 × Hydroxylation + dehydrogenation in dodecane (probably form carboxylic acid at dodecane terminal and one hydroxyl group at dodecane part, or form two hydroxyl group and a keto at dodecane part)
M23	2.31	425,2745	C20H41O9	4 × Hydroxylation + dehydrogenation in dodecane (probably form one extra hydroxyl group at dodecane part of M22)
M24	2.02	441,2694	C20H41O10	5 × Hydroxylation + dehydrogenation in dodecane (probably form one extra hydroxyl group at dodecane part of M23)
M25	1.73	459,2800	C20H43O11	6 × hydroxylation in dodecane (Form six hydroxyl groups at dodecane terminal or part)
M26	1.84	457,2643	C20H41O11	6 × hydroxylation + dehydrogenation in dodecane (Form aldehyde or keto from the hydroxyl group of M25)
M27	1.75	473,2593	C20H41O12	7 × hydroxylation + dehydrogenation in dodecane (probably form one extra hydroxyl group at dodecane part of M26)
M28	1.79	413,2381	C18H37O10	O-dealkylation + 6 × hydroxylation + dehydrogenation in dodecane (Loss of one EO unit (i.e. C2H4O) from the EO groups, and form aldehyde at dodecane terminal and five hydroxyl group at dodecane part or keto and five hydroxyl group at dodecane part)
M29	1.70	429,2330	C18H37O11	O-dealkylation + 7 × hydroxylation + dehydrogenation in dodecane (Form one extra hydroxyl group at dodecane part of M28)
M30	1.73	369,2119	C16H33O9	O-dealkylation (loss of C4H8O2) + 6 × hydroxylation + dehydrogenation in dodecane (Loss of two EO units (i.e. C4H8O2) from the EO groups, and form aldehyde at dodecane terminal and five hydroxyl group at dodecane part or keto and five hydroxyl group at dodecane part)
M31	1.63	385,2068	C16H33O10	O-dealkylation (loss of C4H8O2) + 7 × hydroxylation + dehydrogenation in dodecane (Form one extra hydroxyl group at dodecane part of M30)
M32	1.66	325,1857	C14H29O8	O-dealkylation (loss of C6H12O3) + 6 × hydroxylation + dehydrogenation in dodecane (Loss of three EO units (i.e. C6H12O3) from the EO groups, and form aldehyde at dodecane terminal and five hydroxyl group at dodecane part or keto and five hydroxyl group at dodecane part)
M33	1.56	341,1806	C14H29O9	O-dealkylation (loss of C6H12O3) + 7 × hydroxylation + dehydrogenation in dodecane (Form one extra hydroxyl group at dodecane part of M32)
M34	1.58	281,1595	C12H25O7	O-dealkylation (loss of C8H16O4) + 6 × hydroxylation + dehydrogenation in dodecane (Loss of four EO units (i.e. C8H16O4) from the EO groups, and form aldehyde at dodecane terminal and five hydroxyl group at dodecane part or keto and five hydroxyl group at dodecane part)
M35	1.48	297,1544	C12H25O8	O-dealkylation (loss of C8H16O4) + 7 × hydroxylation + dehydrogenation in dodecane (Form one extra hydroxyl group at dodecane part of M34)

**Table 16:** Metabolite identification with UPLC/QE-orbitrap/MS data for C_16_EO_8_

Metabolite code	Retention time (min)	Calculated m/z	Proposed formula (M + H^+^)	Proposed reaction
C16EO8	4.00	595,4780	C32H67O9	Parent compound
M1	3.25	611,4729	C32H67O10	Hydroxylation in hexadecane (Form hydroxyl group at hexadecane part or terminal)
M2	2.61	644,4943	C32H70NO11	2 × hydroxylation in hexadecane, NH3 adduct (Form one extra hydroxyl group at hexadecane part of M1)
M3	3.17	625,4521	C32H65O11	2 × Hydroxylation + dehydrogenation in hexadecane (Probably form carboxylic acid at hexadecane terminal via omega oxidation or one hydroxyl group at hexadecane terminal and a keto at hexadecane part via omega-1 oxidation)
M4	1.52	371,2276	C16H35O9	O-dehexadecylation (Loss of entire hexadecane (C16H32))
M5	1.53	385,2068	C16H33O10	O-dehexadecylation (loss of C16H32) + hydroxylation + dehydrogenation (probably form carboxylic acid at ethoxy terminal of M4 or form a hydroxyl group ethoxy part and a aldehyde group at ethoxy terminal of M4)
M6	1.48	341,1806	C14H29O9	O-dehexadecylation (loss of C16H32) + O-dealkylation (loss of C2H4O) + hydroxylation + dehydrogenation (Loss of entire hexadecane (C16H32), loss of one EO unit (C2H4O), form carboxylic acid at ethoxy terminal or form a hydroxyl group ethoxy part and a aldehyde group at ethoxy terminal)
M7	1.40	297,1544	C12H25O8	O-dehexadecylation (loss of C16H32) + O-dealkylation (loss of C4H8O2) + hydroxylation + dehydrogenation (Loss of entire hexadecane (C16H32), loss of two EO units (C4H8O2), form carboxylic acid at ethoxy terminal or form a hydroxyl group ethoxy part and a aldehyde group at ethoxy terminal)
M8	1.30	239,1489	C10H23O6	O-dehexadecylation (loss of C16H32) + O-dealkylation (loss of C6H12O3) (Loss of entire hexadecane (C16H32), loss of three EO units (C6H12O3))
M9	1.33	253,1282	C10H21O7	O-dehexadecylation (loss of C16H32) + O-dealkylation (loss of C6H12O3) + hydroxylation + dehydrogenation (probably form carboxylic acid at ethoxy terminal of M8 or form a hydroxyl group at ethoxy part of M8 and a aldehyde group at ethoxy terminal of M8)
M10	1.21	209,1020	C8H17O6	O-dehexadecylation (loss of C16H32) + O-dealkylation (loss of C8H16O4) + hydroxylation + dehydrogenation (Loss of entire hexadecane (C16H32), loss of four EO units (C8H16O4), form carboxylic acid at ethoxy terminal or form a hydroxyl group ethoxy part and a aldehyde group at ethoxy terminal)
M11	1.21	195,1227	C8H19O5	O-dehexadecylation (loss of C16H32) + O-dealkylation (loss of C8H16O4) (Loss of entire hexadecane (C16H32), loss of four EO units (C8H16O4))
M12	4.02	551,4517	C30H63O8	O-dealkylation (Loss one EO unit (C2H4O) of parent compound)
M13	4.03	507,4255	C28H59O7	O-dealkylation (loss of C4H8O2) (Loss two EO units (C4H8O2) of parent compound)
M14	4.04	463,3993	C26H55O6	O-dealkylation (loss of C6H12O3) (Loss three EO units (C6H12O3) of parent compound)
M15	4.04	419,3731	C24H51O5	O-dealkylation (loss of C8H16O4) (Loss four EO units (C8H16O4) of parent compound)
M16	4.05	375,3469	C22H47O4	O-dealkylation (loss of C10H20O5) (Loss five EO units (C10H20O5) of parent compound)
M17	3.99	582,4576	C30H64NO9	O-dealkylation + hydroxylation + dehydrogenation in ethoxy (NH3 adduct) (Loss one EO unit (C2H4O), form carboxylic acid at ethoxy terminal)
M18	4.01	538,4313	C28H60NO8	O-dealkylation (loss of C4H8O2) + hydroxylation + dehydrogenation in ethoxy (NH3 adduct) (Loss two EO units (C4H8O2), form carboxylic acid at ethoxy terminal)
M19	4.03	494,4051	C26H56NO7	O-dealkylation (loss of C6H12O3) + hydroxylation + dehydrogenation in ethoxy NH3 adduct (Loss three EO units (C6H12O3), form carboxylic acid at ethoxy terminal)
M20	4.03	433,3524	C24H49O6	O-dealkylation (loss of C8H16O4) + oxidation + dehydrogenation (Loss four EO units (C8H16O4), form carboxylic acid at ethoxy terminal)
M21	3.18	581,4259	C30H61O10	O-dealkylation + 2 × hydroxylation + dehydrogenation in hexadecane (Loss of one EO unit (C2H4O), form carboxylic acid at hexadecane terminal or form one hydroxyl group at hexadecane part and a aldehyde at hexadecane terminal)
M22	3.18	537,3997	C28H57O9	O-dealkylation (loss of C4H8O2) + 2 × hydroxylation + dehydrogenation in hexadecane (Loss of two EO units (C4H8O2), form carboxylic acid at hexadecane terminal or form one hydroxyl group at hexadecane part and a aldehyde at hexadecane terminal)
M23	3.18	493,3735	C26H53O8	O-dealkylation (loss of C6H12O3) + 2 × hydroxylation + dehydrogenation in hexadecane (Loss of three EO units (C6H12O3), form carboxylic acid at hexadecane terminal or form one hydroxyl group at hexadecane part and a aldehyde at hexadecane terminal)
M24	3.18	449,3473	C24H49O7	O-dealkylation (loss of C8H16O4) + 2 × Oxidation + dehydrogenation in hexadecane (Loss of four EO units (C8H16O4), form carboxylic acid at hexadecane terminal or form one hydroxyl group at hexadecane part and a aldehyde at hexadecane terminal)
M25	3.18	405,3211	C22H45O6	O-dealkylation (loss of C10H20O5) + 2 × Oxidation + dehydrogenation (Loss of five EO units (C10H20O5), form carboxylic acid at hexadecane terminal or form one hydroxyl group at hexadecane part and a aldehyde at hexadecane terminal)
M26	3.18	361,2949	C20H41O5	O-dealkylation (loss of C12H24O6) + 2 × Oxidation + dehydrogenation (Loss of six EO units (C12H24O6), form carboxylic acid at hexadecane terminal or form one hydroxyl group at hexadecane part and a aldehyde at hexadecane terminal)
M27	3.19	612,4317	C30H62NO11	O-dealkylation + 2 × Hydroxylation + dehydrogenation in hexadecane + hydroxylation + dehydrogenation in ethoxy NH3 adduct (Loss of one EO unit (C2H4O), form carboxylic acid at hexadecane terminal or one hydroxyl group and aldehyde at hexadecane terminal, form carboxylic acid at ethoxy terminal)
M28	3.19	568,4055	C28H58NO10	O-dealkylation (loss of C4H8O2) + 2 × Hydroxylation + dehydrogenation in hexadecane + hydroxylation + dehydrogenation in ethoxy NH3 adduct (Loss of two EO units (C4H8O2), form carboxylic acid at hexadecane terminal or one hydroxyl group and aldehyde at hexadecane terminal, form carboxylic acid at ethoxy terminal)
M29	3.19	524,3793	C26H54NO9	O-dealkylation (loss of C6H12O3) + 2 × Hydroxylation + dehydrogenation in hexadecane + hydroxylation + dehydrogenation in ethoxy NH3 adduct (Loss of three EO units (C6H12O3), form carboxylic acid at hexadecane terminal or one hydroxyl group and aldehyde at hexadecane terminal, form carboxylic acid at ethoxy terminal)
M30	3.20	463,3265	C24H47O8	O-dealkylation (loss of C8H16O4) + 2 × Hydroxylation + dehydrogenation in hexadecane + hydroxylation + dehydrogenation in ethoxy NH3 adduct (Loss of four EO units (C8H16O4), form carboxylic acid at hexadecane terminal or one hydroxyl group and aldehyde at hexadecane terminal, form carboxylic acid at ethoxy terminal)
M31	3.19	419,3003	C22H43O7	O-dealkylation (loss of C10H20O5) + 3 × Oxidation + 2 × dehydrogenation (Loss of five EO units (C10H20O5), form carboxylic acid at hexadecane terminal and one hydroxyl group at hexadecane part or two hydroxyl groups and aldehyde at hexadecane terminal or two hydroxyl groups and keto at hexadecane part, form carboxylic acid at ethoxy terminal)
M32	3.19	375,2741	C20H39O6	O-dealkylation (loss of C12H24O6) + 3 × Oxidation + 2 × dehydrogenation (Loss of six EO units (C12H24O6), form carboxylic acid at hexadecane terminal and one hydroxyl group at hexadecane part or two hydroxyl groups and aldehyde at hexadecane terminal or two hydroxyl groups and keto at hexadecane part, form carboxylic acid at ethoxy terminal)
M33	3.79	771,5100	C38H75O15	Glucuronide conjugation (Glucuronidation of parent compound)
M34	3.95	626,4838	C32H68NO10	hydroxylation + dehydrogenation in hexadecane NH3 adduct (Form aldehyde at hexadecane terminal, or keto at hexadecane part)
M35	2.92	597,4208	C30H61O11	O-dealkylation (loss of C2H4O) + 3 × hydroxylation + dehydrogenation in hexadecane (Loss of one EO unit (C2H4O), form carboxylic acid at hexadecane terminal and one hydroxyl group at hexadecane part or two hydroxyl group and aldehyde at hexadecane terminal)
M36	2.66	569,3895	C28H57O11	O-dealkylation (loss of C4H8O2) + 4 × hydroxylation + dehydrogenation in hexadecane (Loss of two EO units (C4H8O2), and form extra one hydroxyl group in M35)
M37	2.40	541,3582	C26H53O11	O-dealkylation (loss of C6H12O3) + 5 × hydroxylation + dehydrogenation in hexadecane (Loss of three EO units (C6H12O3), and form extra one hydroxyl group in M36)
M38	2.14	513,3269	C24H49O11	O-dealkylation (loss of C8H16O4) + 6 × hydroxylation + dehydrogenation in hexadecane (Loss of four EO units (C8H16O4), and form extra one hydroxyl group in M37)
M39	1.89	485,2957	C22H45O11	O-dealkylation (loss of C10H20O5) + 7 × hydroxylation + dehydrogenation in hexadecane (Loss of five EO units (C10H20O5), and form extra one hydroxyl group in M38)
M40	1.71	457,2643	C20H41O11	O-dealkylation (loss of C12H24O6) + 8 × hydroxylation + dehydrogenation in hexadecane (Loss of six EO units (C12H24O6), and form extra one hydroxyl group in M39)
M41	1.58	473,2593	C20H41O12	O-dealkylation (loss of C12H24O6) + 9 × hydroxylation + dehydrogenation in hexadecane (Loss of six EO units (C12H24O6), and form extra one hydroxyl group in M40)
M42	1.72	488,2702	C20H42NO12	O-dealkylation (loss of C12H24O6) + 9 × hydroxylation + 2 × dehydrogenation in hexadecane NH3 adduct (Loss of six EO units (C12H24O6), form carboxylic acid at hexadecane terminal, a keto at hexadecane part and six hydroxyl group at hexadecane part or seven hydroxyl group, a keto and aldehyde at hexadecane terminal)
M43	1.66	413,2381	C18H37O10	O-dealkylation (loss of C14H28O7) + 8 × hydroxylation + dehydrogenation in hexadecane (Loss of seven EO units (C14H28O7), form carboxylic acid at hexadecane terminal and six hydroxyl group at hexadecane part or seven hydroxyl group and aldehyde at hexadecane terminal)
M44	1.53	430,2647	C18H40NO10	O-dealkylation (loss of C14H28O7) + 8 × hydroxylation + dehydrogenation in hexadecane NH3 adduct (Loss of seven EO units (C14H28O7), form carboxylic acid at hexadecane terminal and six hydroxyl group at hexadecane part or seven hydroxyl group and aldehyde at hexadecane terminal)
M45	1.58	429,2304	C18H37O11	O-dealkylation (loss of C14H28O7) + 9 × hydroxylation + dehydrogenation in hexadecane (Loss of seven EO units (C14H28O7), form carboxylic acid at hexadecane terminal and seven hydroxyl group at hexadecane part or eight hydroxyl group and aldehyde at hexadecane terminal)
M46	1.55	444,2424	C18H38NO11	O-dealkylation (loss of C14H28O7) + 9 × hydroxylation + 2 × dehydrogenation NH3 adduct (Loss of seven EO units (C14H28O7), form carboxylic acid at hexadecane terminal, a keto at hexadecane part and six hydroxyl group at hexadecane part or seven hydroxyl group, a keto and aldehyde at hexadecane terminal)
M47	1.68	427,2174	C18H35O11	O-dealkylation (loss of C14H28O7) + 9 × hydroxylation + 2 × dehydrogenation in hexadecane (Loss of seven EO units (C14H28O7), form carboxylic acid at hexadecane terminal, a keto at hexadecane part and six hydroxyl group at hexadecane part or seven hydroxyl group, a keto and aldehyde at hexadecane terminal)
M48	1.60	443,2123	C18H35O12	O-dealkylation (loss of C14H28O7) + 10 × hydroxylation + 2 × dehydrogenation in hexadecane (Loss of seven EO units (C14H28O7), form carboxylic acid at hexadecane terminal, a keto at hexadecane part and seven hydroxyl group at hexadecane part or eight hydroxyl group, a keto and aldehyde at hexadecane terminal)
M49	1.55	399,1861	C16H31O11	O-dealkylation (loss of C16H32O8) + 10 × hydroxylation + 2 × dehydrogenation in hexadecane (Loss of eight EO units (C16H32O8), form carboxylic acid at hexadecane terminal, a keto at hexadecane part and seven hydroxyl group at hexadecane part or eight hydroxyl group, a keto and aldehyde at hexadecane terminal)

**Table 17:** Metabolite identification with UPLC/QE-orbitrap/MS data for C_18_EO_3_

Metabolite code	Retention time (min)	Calculated m/z	Proposed formula (M + H^+^)	Proposed reaction
C18EO3	4.26	403,3782	C24H51O4	Parent compound
M1	3.56	419,3731	C24H51O5	Hydroxylation in octadecane (Form a hydroxyl group at octadecane terminal or part)
M2	3.13	435,3680	C24H51O6	2 × Hydroxylation in octadecane (Form one hydroxyl group at octadecane terminal and one hydroxyl group at octadecane part)
M3	3.60	417,3575	C24H49O5	Hydroxylation + dehydrogenation in octadecane (Form aldehyde (via omega oxidation) or form a keto (omega-1 oxidation) at octadecane part)
M4	4.23	417,3575	C24H49O5	Hydroxylation + dehydrogenation in ethoxy (Form carboxylic acid at ethoxy terminal)
M5	3.46	433,3524	C24H49O6	2 × hydroxylation + dehydrogenation in octadecane (Form carboxylic acid at octadecane terminal or form one hydroxyl group and aldehyde at octadecane terminal)
M6	2.79	449,3473	C24H49O7	3 × hydroxylation + dehydrogenation in octadecane (Form on extra hydroxyl group at octadecane part of M5)
M7	3.47	447,3316	C24H47O7	3 × Oxidation + 2 × dehydrogenation (Form carboxylic acid at octadecane terminal and a keto at octadecane part, or form aldehyde at octadecane terminal, a keto and a hydroxyl group at octadecane part)
M8	4.13	401,3625	C24H49O4	Dehydrogenation in octadecane (Form a double bond at octadecane part)
M9	3.09	595,4052	C30H59O11	Hydroxylation in octadecane + glucuronide conjugation (Glucuronidation of M1)
M10	2.58	611,4001	C30H59O12	2 × Hydroxylation in octadecane + glucuronide conjugation (Glucuronidation of M2)
M11	3.06	626,4110	C30H60NO12	2 × Hydroxylation + dehydrogenation in octadecane + glucuronide conjugation (NH3 adduct) (Glucuronidation of M5)
M12	3.07	419,3731	C24H51O5	Hydroxylation in ethoxy (Form a hydroxyl group at ethoxy part)
M13	3.17	450,3789	C24H52NO6	2 × hydroxylation + dehydrogenation NH3 adduct (Form carboxylic acid at octadecane terminal or form one hydroxyl group and aldehyde at octadecane terminal)
M14	2.84	612,4317	C30H62O11	Hydroxylation + glucuronide conjugation NH3 adduct (Glucuronidation of M1)
M15	3.17	405,3211	C22H45O6	O-dealkylation (loss of C2H4O) + 3 × hydroxylation + dehydrogenation (Loss of one EO unit (C2H4O) from EO groups, form carboxylic acid and one hydroxyl group at octadecane part, or form a aldehyde and two hydroxyl groups at octadecane part, or carboxylic acid at ethoxy terminal and two hydroxyl groups at octadecane part)
M16	2.90	377,2898	C20H41O6	O-dealkylation (loss of C4H8O2) + 4 × hydroxylation + dehydrogenation (Loss of two EO units (C4H8O2) from EO groups, form carboxylic acid and two hydroxyl groups at octadecane part, or form a aldehyde and three hydroxyl groups at octadecane part, or carboxylic acid at ethoxy terminal and three hydroxyl groups at octadecane part)
M17	2.61	349,2585	C18H37O6	O-dealkylation (loss of C6H12O3) + 5 × hydroxylation + dehydrogenation (Loss of all EO groups, form carboxylic acid and three hydroxyl groups at octadecane part, or form a aldehyde and four hydroxyl groups at octadecane part)
M18	2.23	365,2534	C18H37O7	O-dealkylation (loss of C6H12O3) + 6 × hydroxylation + dehydrogenation (Loss of all EO groups, form carboxylic acid and four hydroxyl groups at octadecane part, or form a aldehyde and five hydroxyl groups at octadecane part)

**Table 18:** Representative constitute and SMILES codes

Constituent	SMILES codes	Number of metabolites			
		Hydrolysis (Acidic)	In vivo Rat	Rat liver S9	Skin metabolism
C_8_EO_4_	CCCCCCCCOCCOCCOCCOCCO	8	45	22	2
C_10_EO_5_	CCCCCCCCCCOCCOCCOCCOCCOCCO	10	56	30	1
C_12_EO_4_	CCCCCCCCCCCCOCCOCCOCCOCCO	8	51	22	1
C_16_EO_8_	CCCCCCCCCCCCCCCCOCCOCCOCCOCCOCCOCCOCCOCCO	16	78	27	1
C_18_EO_3_	CCCCCCCCCCCCCCCCCCOCCOCCOCCO	6	43	14	1

## Appendix 2

See Figs. 5, 6, 7, 8, 9, 10, 11, 12, 13, 14, 15, 16, 17, 18, 19, 20, 21, 22, 23 and 24.
